# Folic Acid Exposure Rescues Spina Bifida Aperta Phenotypes in Human Induced Pluripotent Stem Cell Model

**DOI:** 10.1038/s41598-018-21103-8

**Published:** 2018-02-13

**Authors:** Vardine Sahakyan, Robin Duelen, Wai Long Tam, Scott J. Roberts, Hanne Grosemans, Pieter Berckmans, Gabriele Ceccarelli, Gloria Pelizzo, Vania Broccoli, Jan Deprest, Frank P. Luyten, Catherine M. Verfaillie, Maurilio Sampaolesi

**Affiliations:** 1 Translational Cardiomyology Laboratory, Stem Cell Biology and Embryology Unit, Stem Cell Institute, Department of Development and Regeneration, KU Leuven, Leuven, Belgium; 2 Tissue Engineering Laboratory, Skeletal Biology and Engineering Research Center, and Prometheus, Division of Skeletal Tissue Engineering, KU Leuven, Leuven, Belgium; 3 Institute of Orthopaedics and Musculoskeletal Science, Division of Surgery and Interventional Science, University College London, The Royal National Orthopaedic Hospital, London, UK; 4 Stem Cell Institute and Stem Cell Biology and Embryology Unit, Department Development and Regeneration, KU Leuven, Leuven, Belgium; 5 Human Anatomy Unit, Department of Public Health, Experimental and Forensic Medicine, University of Pavia, Pavia, Italy; 6 Pediatric Surgery Department, Istituto Mediterraneo di Eccellenza Pediatrica (ISMEP), Children’s Hospital “G di Cristina”, Palermo, Italy; 7 Stem Cell and Neurogenesis Unit, Division of Neuroscience, San Raffaele Scientific Institute, Milan, Italy; 8 CNR-Institute of Neuroscience, Milan, Italy; 9 Department of Obstetrics and Gynecology, Division Woman and Child, Fetal Medicine Unit, University Hospitals KU Leuven, Leuven, Belgium; 10 Institute for Women’s Health (IWH), University College London, London, United Kingdom

## Abstract

Neural tube defects (NTDs) are severe congenital abnormalities, caused by failed closure of neural tube during early embryonic development. Periconceptional folic acid (FA) supplementation greatly reduces the risk of NTDs. However, the molecular mechanisms behind NTDs and the preventive role of FA remain unclear. Here, we use human induced pluripotent stem cells (iPSCs) derived from fetuses with spina bifida aperta (SBA) to study the pathophysiology of NTDs and explore the effects of FA exposure. We report that FA exposure in SBA model is necessary for the proper formation and maturation of neural tube structures and robust differentiation of mesodermal derivatives. Additionally, we show that the folate antagonist methotrexate dramatically affects the formation of neural tube structures and FA partially reverts this aberrant phenotype. In conclusion, we present a novel model for human NTDs and provide evidence that it is a powerful tool to investigate the molecular mechanisms underlying NTDs, test drugs for therapeutic approaches.

## Introduction

Neural tube defects (NTDs) are a common type of congenital anomaly. The manifestation of NTDs occurs in very early stages of embryonic development, with failed closure of the neural tube at around day 28^1^. An occurrence frequency of the disease is 1 case per 1,000 births^2,3^ and each year nearly 300,000 infants with NTDs are born, further resulting in death or lifelong disabilities^4^. Therefore, socioeconomic cost associated with NTD patients is very high due to the increased morbidity and premature mortality^5^.

Spina bifida aperta (SBA) is one of the most severe types of NTDs associated with herniation of neural tissue through an incompletely formed spine. SBA is a progressive, nonlethal but yet chronic disease with significant morbidity^6,7^. The condition can be easily picked up in first trimester screening programs. However, in practice, most diagnoses are still made in the second trimester^8^. Although fetal surgery by prenatal repair of the damage is a common treatment approach for SBA^8^, the malformation may lead to severe progressive complications after birth, like hydrocephalus, cognitive impairments, and sensory-motor deficits^6,7^.

The aetiology of SBA, and NTDs in general, is poorly understood^9,10^. Preclinical studies performed on mice showed numerous genes associated with the disease; however, specific genes described in mice are not sufficient to explain the heterogeneity of NTDs in humans^1,9^. Over 25 years of clinical and experimental studies indicate that NTDs arise from a combination of genetic and gene-environment interaction factors.

The risk of NTDs is greatly reduced by folic acid (FA) taken as a supplement starting from at least one month before conception and continuing throughout the first trimester of pregnancy^1,11^. The folate metabolic pathway plays a crucial role in nucleotide biosynthesis, proper cell proliferation and generation of methyl donors^12–14^. Moreover, during the first-trimester of pregnancy, exposure to FA antagonists is associated with an increased risk of congenital anomalies, including NTDs^15^. For instance, exposure to the folate antagonist methotrexate (MTX) induces NTDs in animal models^16^. MTX inhibits dihydrofolate reductase (DHFR) an enzyme that participates in the tetrahydrofolate (THF) synthesis from folate^17^. However, the exact mechanism through which MTX causes and FA supplementation prevents NTDs remains unknown^1^.

High and persisting proliferation of neural stem cells (NSCs) is required for the normal development and correct morphogenesis of the central nervous system (CNS). FA, influences the proliferation and differentiation of NSCs, whereas MTX impairs cell proliferation of embryonic NSCs in animal models^18^. Consistent folate deficiency can also lead to various neurological conditions in children and adults^19,20^. Folate transport through its own receptors might be crucial to prevent NTDs, as, for instance, Folate Receptor 1 (*FOLR1*, a member of the family of folate transport genes) is involved in the maintenance of intracellular folate level^21^, and, mice lacking Folr1 exhibited failure of neural tube closure^22^. *FOLR1* receptors are expressed on the plasmatic membrane of the human placenta^23^, where they play a role in folate transport during early embryonic development. The mechanistic link between *FOLR1* and NTDs is also unclear. Thus, *in vitro* models of human NTDs to capture the pathological phenotype and reveal the mechanism of FA action are urgently needed.

Human induced pluripotent stem cell (iPSC) technology could provide an attractive model to recapitulate the disease with its specific mechanisms and address early events in NTD manifestation. During the early neural differentiation, pluripotent stem cells (PSCs) undergo morphogenetic events and form radially arranged columnar epithelial cells, named neural rosettes. Neural rosettes resemble the structure of embryonic neural tube and express several early neural and radial glia (RG) markers, including PAX6, SOX1, NESTIN, BLBP (known also as FABP7) similar to the developing neural tube^24–26^. Cells within rosettes acquire apical-basal polarity based on the localized expression of adherence and tight junction proteins, including N-CAD and ZO-1 in a ring surrounding the central lumen^27,28^. The structures, proliferation potential and proper polarization of the neural rosettes are significant features for their neurogenic potential^25,27^. Thus, generation of the neural rosettes from iPSCs derived from NTD patients and determination of the disease features and mechanisms are of utmost importance to allow intervention strategies to be proposed.

Musculoskeletal problems are common among SBA patients and in animal models^29,30^, confirming the complexity of the disease and its involvement in various tissues. Several genes associated with NTDs, including Pax3^31,32^ in mice, play an important role in mesodermal derivatives and loss of mesoderm in zebrafish leads to NTDs^33^. Thus, the exploration of the human NTD characteristics in mesodermal derivatives is timely.

In the present study, we established a human NTD cell model using disease-specific iPSCs. We described the morphological features of the disease administrated or not with FA during early neural and mesodermal lineages specification. We also explored the effect of the folate antagonist MTX during SBA iPSC differentiation towards neural rosettes.

## Results

### Generation of SBA and healthy iPSCs using non-integrating Sendai viral vectors

To avoid transgene insertion into the host genome, we used a non-integrating Sendai virus (SeV) reprogramming approach to generate iPSCs from skin fibroblasts derived from 3 SBA fetuses and from 1 amniotic fluid derived stem cells (AFSCs) sample. In addition, we generated iPSCs from skin fibroblasts of 2 healthy adult donors and from 1 AFSC sample derived from healthy fetus as controls. Reprogramming efficiency by SeV was 0.07% as previously reported^34^ and we were able to generate several iPSC lines from each fetus and healthy donor samples. We deeply characterized 3 lines derived from each fetus and healthy donors.

**Figure 1:**
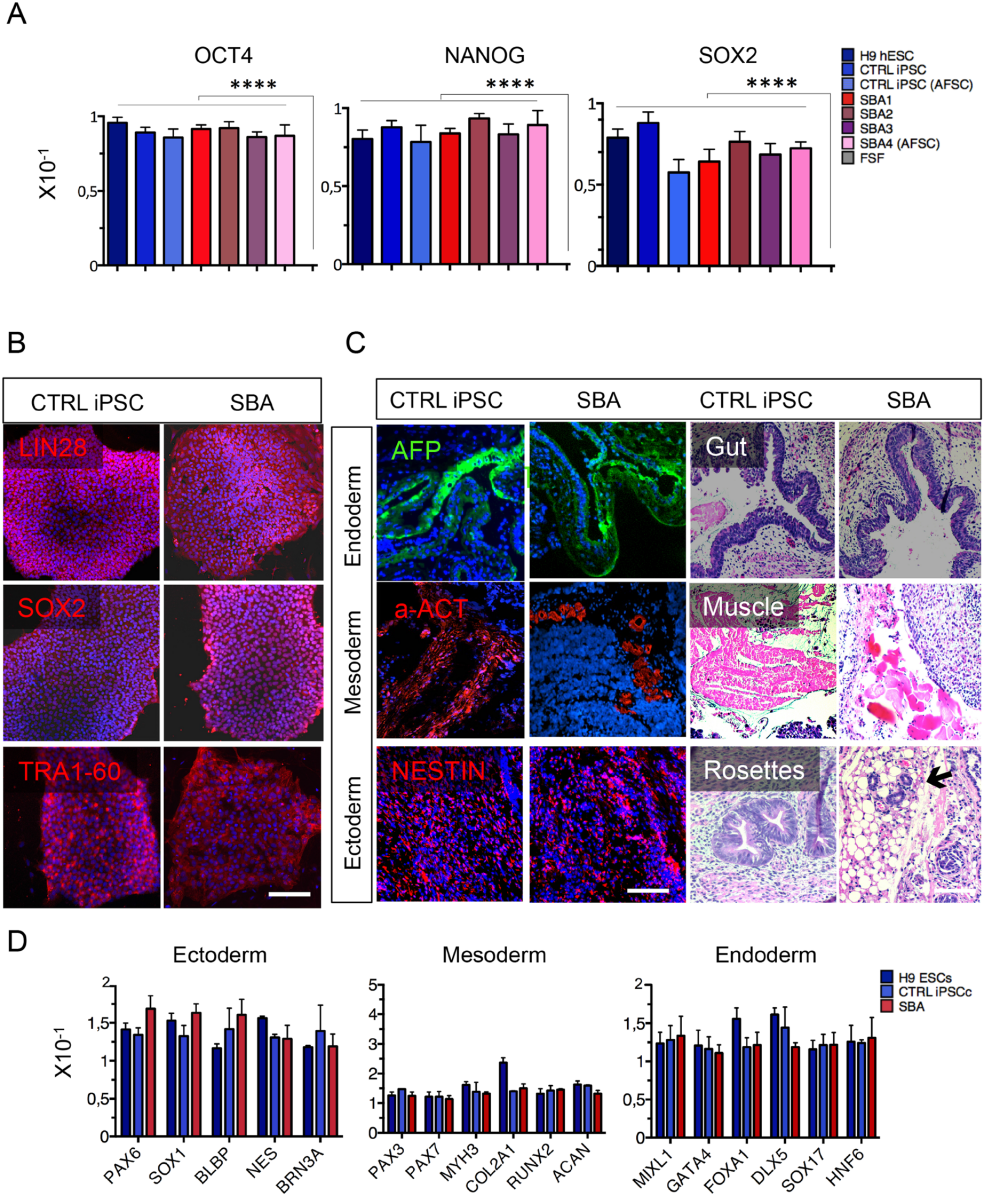
Generation of human iPSCs derived from SBA fetuses. (**A**) qRT-PCR data from CTRL iPSC and SBA lines for pluripotency markers *OCT4, NANOG, SOX2* are shown as relative expression to housekeeping gene *GAPDH*. H9 human ESCs and FSFs were respectively used as a positive and negative control; Data are plotted as average ± SEM. ****P < 0.0001 H9 hESCs, CTRL iPSCs, CTRL iPSC (AFSC), SBA1, SBA2, SBA3, SBA4 (AFSC) *vs* FSF. N = 4 independent experiments per iPSC line. (**B**) IF analysis for pluripotency markers SOX2, LIN28 and TRA-1–60 (all in red) in CTRL iPSC and SBA lines. Representative images of CTRL1#2 and SBA2#1 are shown. Nuclei were counterstained in blue with DAPI; scale bar = 100 μm. (**C**) Teratomas with presence of derivatives of all three-germ layers generated from CTRL iPSC and SBA lines, as illustrated by presence of endoderm: AFP and gut, mesoderm: αSMA and muscle and ectoderm: NESTIN and neural rosettes. Representative images of CTRL1#2 and SBA2#1 are shown; scale bar = 200 μm. (**D**) qRT-PCR data for the genes representing three-germ layers on H9 ESC, CTRL iPSC and SBA-derived teratomas. Ectodermal (*PAX6, SOX1, BLBP, NES, BRN3A)*, mesodermal (*PAX3, PAX7, MYH3, COL2A1, RUNX2, ACAN)* and endodermal *(MIXL1, GATA4, FOXA1, DLX5, SOX17, HNF6)* markers are shown as relative expression to housekeeping gene *GAPDH*. Data are plotted as average ± SEM. N = 4 independent experiments per iPSC line.

qRT-PCR analysis for PSC markers (*OCT4*, *NANOG, SOX2*) was performed in SeV generated iPSCs from SBA and healthy donors (Fig. 1A). Additionally, human embryonic stem cells (ESCs) and fetal skin fibroblasts (FSFs) were used as positive and negative controls respectively. All iPSC lines showed pluripotent gene expression similar to human ESCs. In addition, the expression of this class of genes was maintained unaltered at early and late passages (8–25 passages) of all iPSC lines (data not shown). Consistently, *SOX2, LIN28*, and *TRA1*-60 gene products showed correct levels and sub-cellular localization when detected in iPSCs by immunofluorescence (IF) (Fig. 1B). All iPSCs were able to form teratoma in immunodeficient mice in 5–8 weeks after grafting (Fig. 1C). We also performed qRT-PCR analysis and confirmed the presence of three germ layers in teratoma tissues generated by SBA-, healthy iPSCs and human ESCs (Fig. 1D).

Finally, to assess the functional pluripotency and *in vitro* differentiation potential of human iPSCs, we used spontaneous differentiation and TaqMan^®^ hPSC Scorecard™ panel comprehensive gene expression real-time PCR assay^35,36^. Our results revealed that majority of iPSC lines, generated both from SBA and healthy donors were pluripotent and able to differentiate toward all three germ layers (Supplementary Fig. S1A). However, 2 SBA and 1 healthy-derived iPSC lines were excluded from our study, since they demonstrated lower level of pluripotency genes expression and inadequate differentiation capacity towards three-germ layers (Supplementary Fig. S1B). More details about iPSC lines used in this study are presented in Supplementary Information (Supplementary Note).

Taken together, our results showed that SeV-generated SBA and healthy iPSCs exhibited similar characteristics to human ESCs, with a great ability to differentiate toward derivatives of all three germ layers.

### Aberrant number and size of neural rosettes derived from SBA iPSCs

To assess neural tube-like structures formation potential of SBA iPSCs, we induced *in vitro* neural differentiation and rosette formation. We generated neural rosettes, widely used procedure to mimic early neural tube and CNS structures, from all PSCs. To test whether FA supplementation had any role on NSC induction and neural rosette formation; we performed all experiments with and without FA administration. Dose dependent experiments showed that additional 4.5 μM FA was a suitable dose for neural induction, since it did not affect PSCs and NSCs viability (Supplementary Fig. S2A–C). We added additional 4.5 μM FA to the culture medium during the first 12 days of NSC induction from all PSC, before the formation of neural rosettes. All iPSC lines maintained PSC morphology and showed similar growth and proliferation potential before the neural induction. During the neural induction, all cell lines changed morphology and formed tightly crowded neuroepithelial (NE) cell layers with small nuclei in all conditions (Supplementary Fig. S3A). No differences were observed in morphology and proliferation and the initial immature rosettes started to appear at day 14 from neural induction in all samples. The rosettes from SBA lines were smaller and in higher number compared to controls. We stained rosettes nuclei with DAPI and confirmed these findings (Supplementary Fig. S3B panels left). Interestingly, FA exposed SBA cells generated larger rosettes with respect to those obtained by SBA untreated cells and more comparable to the structures originated by control cells in normal conditions (Supplementary Fig. S3B panels right). Subsequently, we measured the diameter size of rosettes derived from SBA and control lines and confirmed that the majority of SBA rosettes were significantly smaller *(p* < *0.0001)* and the diameter size was between 40–70 μm while in the controls the majority of rosette diameter size was more than 100 μm. FA exposure significantly increased the SBA neural rosette diameter sizes *(p* < *0.0001)* that reached more than 100 μm. FA exposure did not affect on control-derived neural rosettes diameter sizes (Supplementary Fig. S3C). Additionally, we counted the rosette number derived from all cell lines and confirmed that SBA lines compared to controls generated significantly more rosettes *(p* < *0.0001)*. Interestingly, upon FA exposure the number of SBA derived rosettes decreased and became similar to that in controls, and no significant differences were found in FA exposed control rosettes (Supplementary Fig. S3D).

Taken together, our data demonstrated that SBA iPSCs formed aberrant neural tube structures, presented smaller rosette diameter sizes and in higher quantities as compared to controls. Additionally, FA exposure rescued both aberrant phenotypes, suggesting a critical role of FA in neural rosette formation.

### No alterations in early neural gene expressions and protein localization in SBA fetal iPSC-derived NSCs

**Figure 2:**
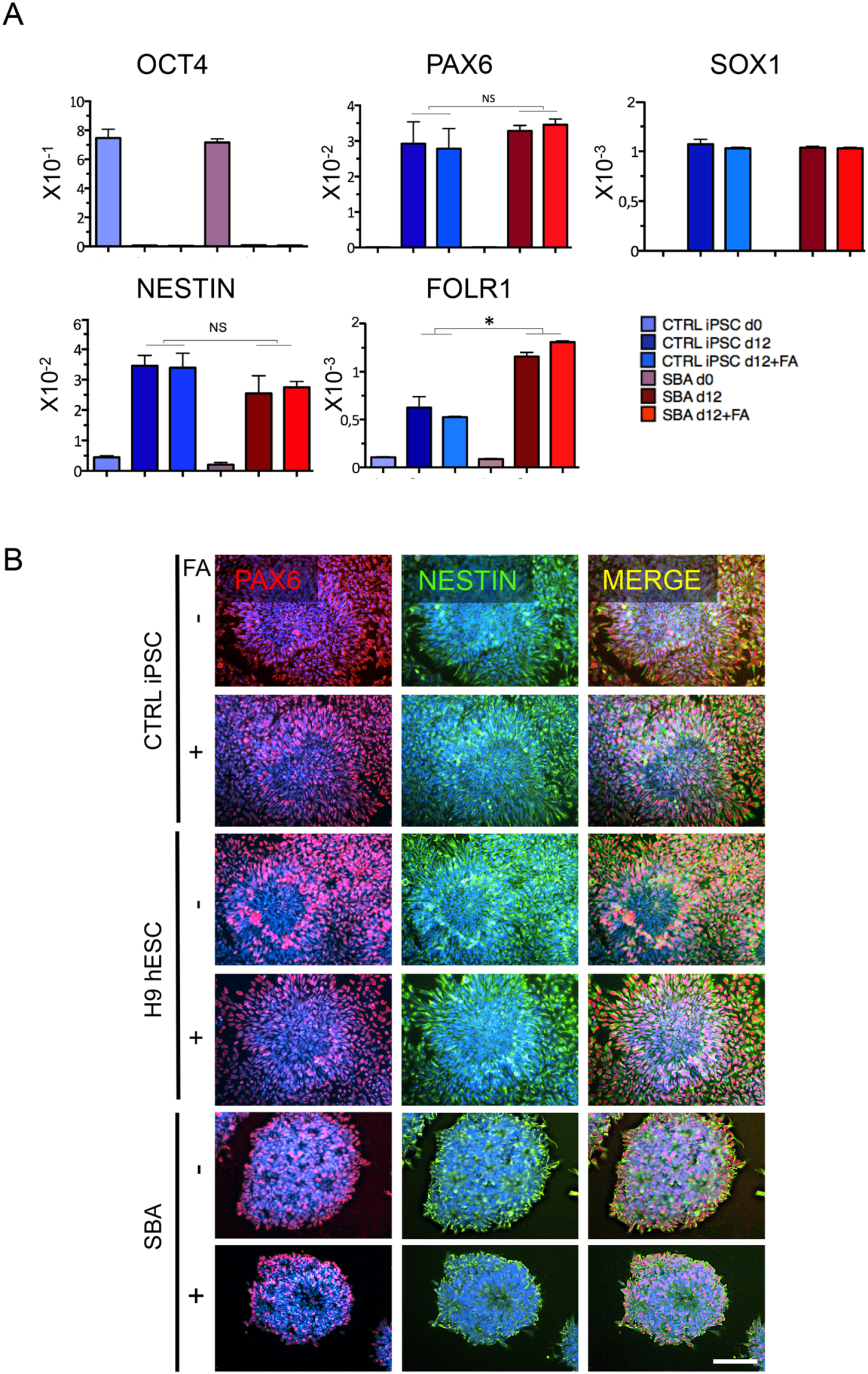
Early neural gene expressions and protein localization in SBA iPSC-derived NSCs. (**A**) qRT-PCR data of CTRL iPSC and SBA-derived NSCs for pluripotency (*OCT4)*, early neural (*PAX6*, *SOX1*, *NESTIN*) and folate receptor 1 (*FOLR1)* genes at day 0 and day 12 of neural differentiation are shown as relative expression to housekeeping gene *GAPDH*. NS, not significant. Data are plotted as average ± SEM. *P < 0.05 CTRL iPSC d12 *vs* SBA d12 and CTRL iPSC + FA d12 *vs* SBA + FA d12 (*FOLR1*). N = 4 independent experiments per iPSC line. See also Supplementary Fig. S4A (**B**) IF staining for early neural markers PAX6 (red) and NESTIN (green) in CTRL iPSC, H9 hESC and SBA-derived neural rosettes at day 18 of neural differentiation. Representative images of CTRL1#2 and SBA2#1 are shown. Nuclei were counterstained with DAPI (blue). Scale bar = 100 μm.

To test whether NSCs derived from SBA lines exhibited any peculiar expression of early neural genes, we next assessed the levels of early neural markers (*PAX6, SOX1* and *NESTIN)* and the Folate Receptor 1 (encoded by the *FOLR1* gene) at day 0 and day 12 of neural differentiation. An upregulation of the early neural markers in concomitance with a parallel decrease of the pluripotency marker *OCT4* was observed in all SBA samples at day 12 of differentiation similar to the controls. Early neural markers were not affected by additional FA exposure. Surprisingly, we found that *FOLR1* was significantly upregulated in SBA lines compared to the controls, which was also independent from FA exposure (Fig. 2A and Supplementary Fig. S4A).

Additionally, at day 18 of neural induction we assessed early neural markers localization in the rosettes. IF analysis indicated that columnar cells in SBA iPSC-derived rosettes were double positive for PAX6 and NESTIN, similar to the controls, independent from FA exposure (Fig. 2B).

In summary, despite the small size and increased numbers, SBA-derived neural rosettes appeared to correctly express a set of early neural markers, and more FA did not altered the overall expression of neural fate cardinal markers. However, FOLR1 was significantly overexpressed in SBA lines compared to the controls, most probably in an attempt to increase folate signaling in SBA NSCs.

### Correct polarization of neural rosettes derived from SBA iPSCs

**Figure 3:**
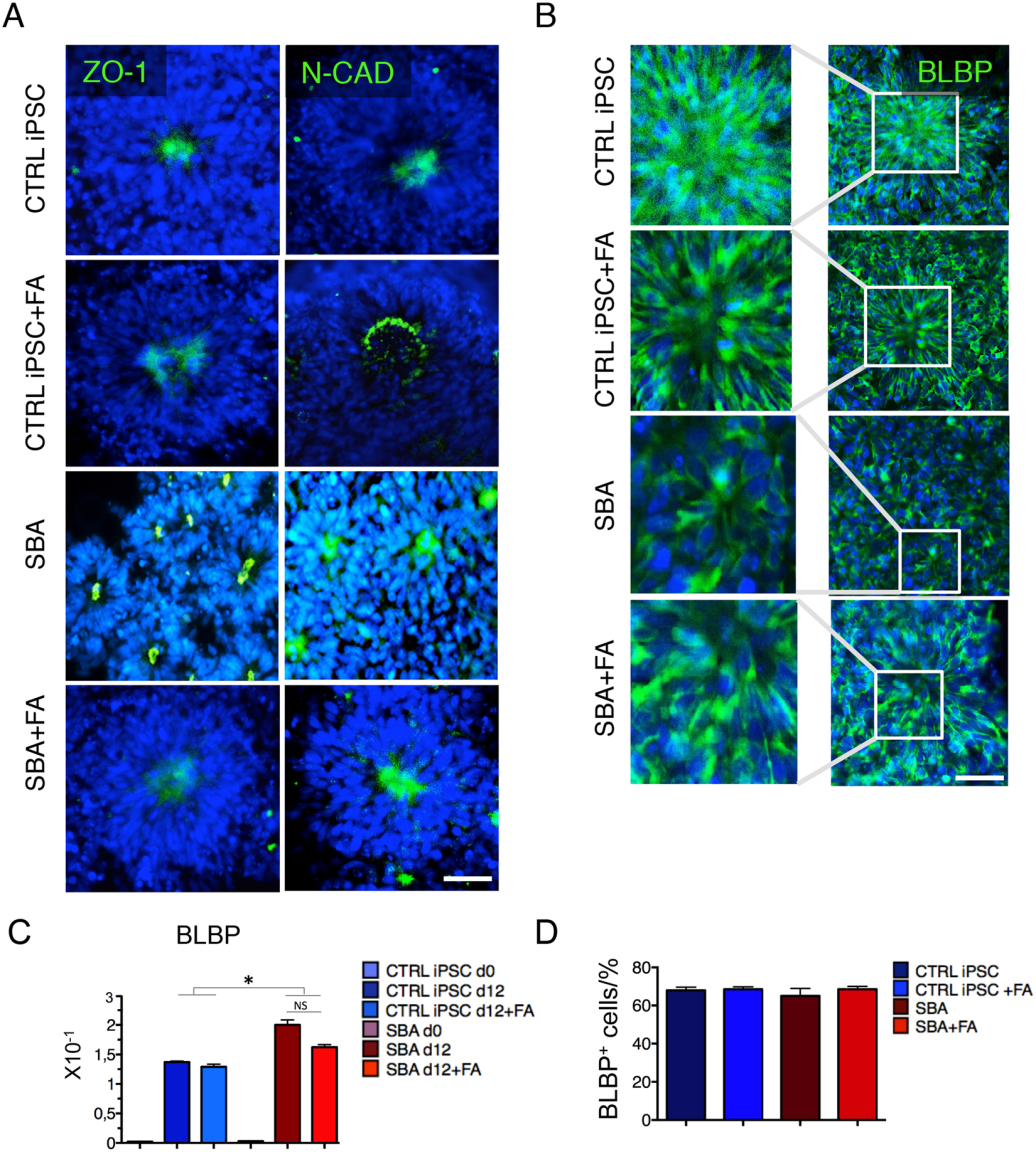
Polarization and radial-glia commitment of SBA iPSC-derived neural rosettes. (**A**) IF staining for adherence and tight junction protein markers ZO-1 and N-CAD (both in green) in CTRL iPSC and SBA-derived neural rosettes at day 18 of neural differentiation. Representative images of CTRL1#2 and SBA2#1 are shown. Nuclei were counterstained with DAPI (blue). Scale bar = 100 μm. See also Supplementary Fig. S4B. (**B**) IF staining analysis for BLBP (green) in CTRL iPSC and SBA-derived neural rosettes at day 18 of neural differentiation. Representative images of CTRL1#2 and SBA2#1 are shown. Nuclei were counterstained with DAPI (blue). Scale bar = 100 μm. (**C**) qRT-PCR data for early RG marker *BLBP* at day 0 and 12 from neural differentiation in CTRL iPSC and SBA-derived NSCs shown as relative expression to housekeeping gene *GAPDH*. NS, not significant. Data are plotted as average ± SEM. *P < 0.05 CTRL iPSC d12 *vs* SBA d12 and CTRL iPSC + FA d12 *vs* SBA + FA d12. N = 4 independent experiments per iPSC line. (**D**) Enumeration of BLBP+ cells in CTRL iPSC and SBA-derived neural rosettes at day 18 of neural differentiation shown as percentage. Data are plotted as average ± SEM. N = 4 independent experiment per iPSC line.

Polarity of NSCs within rosettes is one of the most important aspects for neural tube development and one of the key features for correct dynamics of NSCs proliferation and differentiation^25^. To this end, we investigated the localization of crucial cell polarity cues within the rosette forming cells. In particular, we evaluated the apical localization of Zona occludens-1 (ZO-1) (known also as Tight junction protein 1,TJP1) and basal enrichment of the adherent junction’s marker N-cadherin (N-CAD) at day 18 of differentiation of SBA iPSCs-derived neural rosettes. Both proteins were localized on the inward side facing the central lumen of the rosettes in all cell types as expected. Additionally, FA exposure maintained correct polarization in all samples (Fig. 3A and Supplementary Fig. S4B).

In brief, neural rosettes derived from SBA lines displayed a correct apical–basal polarity during rosette formation and FA exposure provided rosette structure alterations without affecting cell polarity.

### Commitment to radial glia of SBA iPSCs

To evaluate RG commitment from SBA derived NSCs, we investigated the expression of the RG marker *BLBP* by qRT-PCR at day 0 and day 12 of neural differentiation. *BLBP* was significantly upregulated in SBA lines compared to the controls independently from FA exposure (Fig. 3C). We also checked the sub-cellular localization of the BLBP protein in neural rosettes, by performing IF analysis at day 18 of neural induction, however, with no significant differences observed (Fig. 3B,D).

In summary, SBA iPSCs showed RG commitment and correct BLBP protein localization, though a statistically significant upregulation of *BLBP* was observed independently from FA exposure.

### Impairment of Ki67+ cells in neural rosettes derived from SBA iPSCs and rescue through additional FA administration

We next investigated whether the small size and increased number of SBA iPSC-derived rosettes was associated to any impairment in cell proliferation. We checked Ki67 (also known as MKI67), marker strictly associated with cell proliferation, at day 6 of neural induction. We found that the frequency of Ki67+ cells in SBA iPSC and control lines progeny were similar (around 92%), independently from FA administration. In addition, upon FA exposure, Ki67+ cells significantly increased *(p* < *0.0001)* in all cell lines (around 98%) (Supplementary Fig. S5A,B). We then determined Ki67 localization in neural rosettes. IF analysis indicated that at day 18 of neural induction Ki67+ cells were decreased in all cell lines as compared to day 6 of the neural induction. Surprisingly, SBA rosettes presented significantly less Ki67+ cells compared to the controls *(p* < *0.0001)*. Moreover, several SBA rosettes were entirely Ki67 negative, indicative of an absence of proliferative cells. Importantly, FA exposure significantly increased *(p* < *0.0001)* the number of Ki67+ cells in SBA rosettes similar to the controls values (Supplementary Fig. S5C,D).

In conclusion, during early neural induction, expression of Ki67 was similar in SBA and control lines, and FA exposure similarly increased the number of Ki67+ cells in all cell lines. However, at late stage, Ki67+ cells were significantly reduced in SBA derived neural rosettes, as compared to the controls. FA exposure could rescue this phenotype suggesting an important role of FA in NSCs proliferation.

### 3D spheroids derived from SBA iPSCs represent aberrant structures and generate less early neurons, rescued by FA exposure

**Figure 4:**
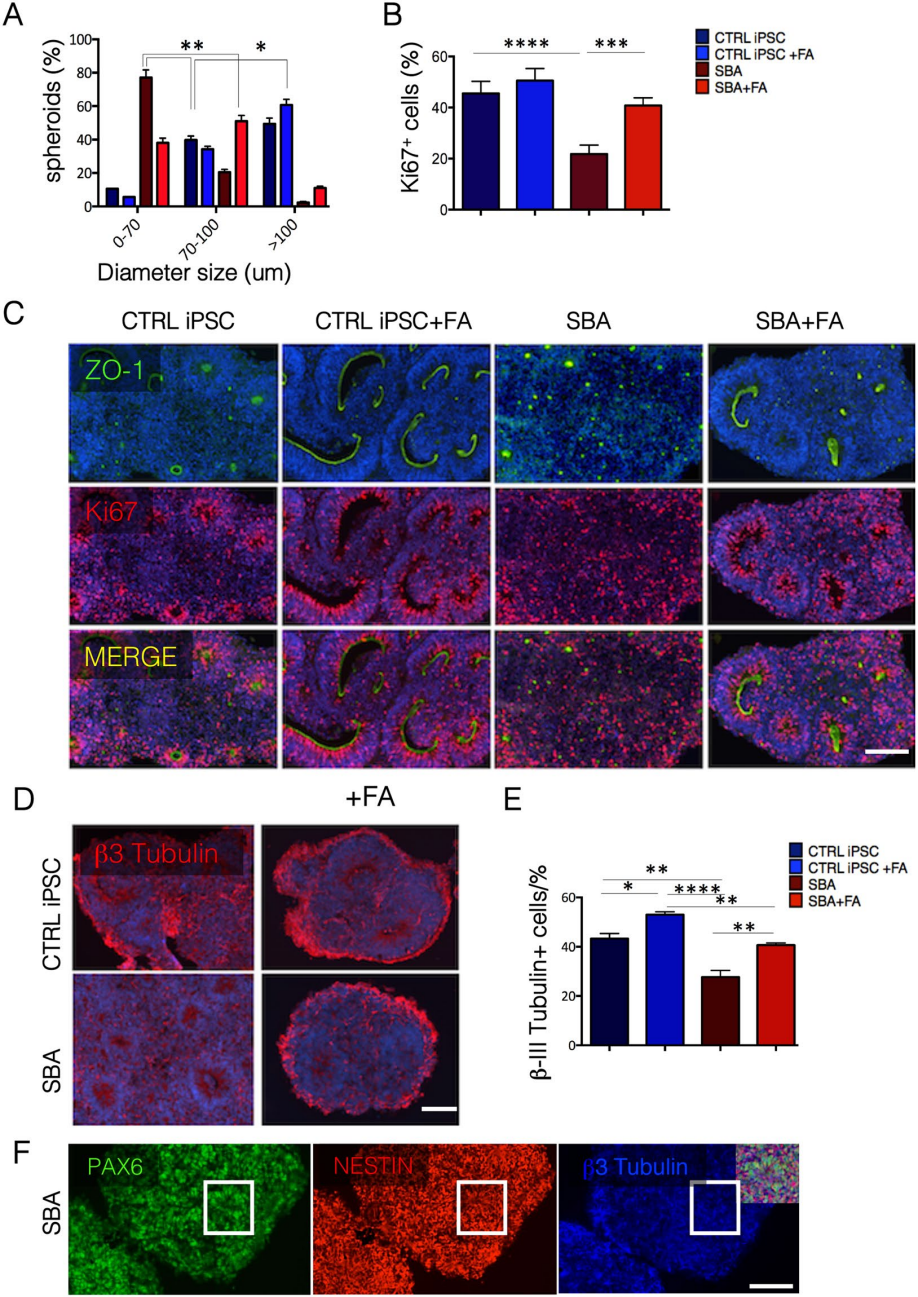
Characterization of 3D spheroids, generated from SBA iPSC-derived neural rosettes. (**A**) Diameter size (μm) of CTRL iPSC and SBA neural rosettes-derived 3D spheroids shown as percentage. Data are plotted as average ± SEM. **P < 0.01 CTRL iPSC *vs* SBA and SBA *vs* SBA + FA, *P < 0.05 CTRL iPSC *vs* CTRL iPSC + FA. N = 4 independent experiment per iPSC/hESC line. (**B**) Enumeration of Ki67+ cells in CTRL iPSC and SBA neural rosettes-derived 3D spheroids shown as percentage. NS, not significant. Data are plotted as average ± SEM ****P < 0.0001 CTRL iPSC *vs* SBA, SBA *vs* SBA + FA, ***P < 0.001 CTRL iPSC *vs* CTRL iPSC + FA. N = 4 independent experiment per iPSC line. (**C**) IF staining for tight junction protein marker ZO-1 (green) and Ki67 (red) in CTRL iPSC and SBA-derived 3D spheroids sections. Representative images of CTRL iPSC1#2 and SBA2#1 are shown. Nuclei were counterstained with DAPI (blue). Scale bar = 200 μm for ZO-1, 100 μm for Ki67. (**D**) IF staining for early neural differentiation marker β–III Tubulin (red) in CTRL iPSC SBA neural rosettes-derived 3D spheroids sections. Nuclei were counterstained with DAPI (blue). Representative images of CTRL iPSC1#2 and SBA2#1 are shown. Scale bar = 200 μm. (**E**) Enumeration of β–III Tubulin+ cells in CTRL iPSC and SBA neural rosettes-derived spheroids shown as percentage. Data are plotted as average ± SEM. *P < 0,05 CTRL *vs* CTRL + FA, **P < 0.01 CTRL iPSC *vs* SBA, CTRL iPSC + FA *vs* SBA + FA, SBA *vs* SBA + FA ****P < 0.0001 CTRL iPSC + FA *vs* SBA. N = 4 independent experiment per line. (**F**) IF staining for PAX6 (green), NESTIN (red) and β–III Tubulin (blue) in SBA neural rosettes-derived 3D spheroids sections. Representative images of SBA2#1 are shown. Scale bar = 200 μm.

We next generated 3D spheroids from neural rosettes to enhance the maturation of the neural tube-like structures (Supplementary Fig. S6). At day 7, paraffin-embedded spheroids sections were characterized for their structures, polarization and proliferation potential. The 3D spheroids from SBA iPSC lines were significantly smaller as compared to the controls and FA exposure significantly rescued the small size (Fig. 4A). All spheroids displayed correct polarization by expressing ZO-1 in the lumen (Fig. 4C panels top-left). Notably, FA exposure improved the structure of the SBA and control-derived spheroids, which presented elongated neural tube structures, better resembling the *in vivo* neural tube structures (Fig. 4C panels top-right).

We determined if 3D spheroids derived from SBA lines were proliferating less similarly to SBA iPSC-derived neural rosettes. Indeed, we found that the amount of Ki67+ cells was significantly less in SBA-derived spheroids as compared to the controls. FA exposure significantly improved the fraction of Ki67+ cells in SBA-derived spheroids similarly to the controls (Fig. 4B,C panels central).

To evaluate early neural differentiation potential, we stained spheroids sections with the β-III Tubulin. Surprisingly, we found that SBA lines generated less β-III Tubulin+ cells compared to the controls and FA exposure greatly increased the number of β-III Tubulin+ cells in SBA and control derived spheroids (Fig. 4D,E). Nevertheless, the majority of cells were double positive for PAX6 and NESTIN (Fig. 4F).

These data suggest that the SBA-derived 3D spheroids displayed similar characteristics (smaller diameter size and less Ki67+ cells) to neural rosettes and further maturation did not affect their size. Moreover, the amount of early-generated neurons was significantly reduced in SBA-derived 3D spheroids compared to controls. Finally, FA exposure rescued this aberrant phenotype and greatly increased the amount of β-III Tubulin+ cells in all spheroids, underlining the critical role of FA in early neural differentiation process.

### Ablation of neural rosette formation by folate antagonist MTX and rescue by FA exposure

We next explored NSCs and rosette formation in the presence of folate antagonist MTX. A dose dependence assay was performed and 0,22 μM of MTX found to be an optimal dose for neural differentiation experiments, since it did not affect cell viability (data not shown). MTX was added to the culture medium only during the first three days of neural induction with or without FA exposure. FA was added during the whole period of neural induction.

**Figure 5:**
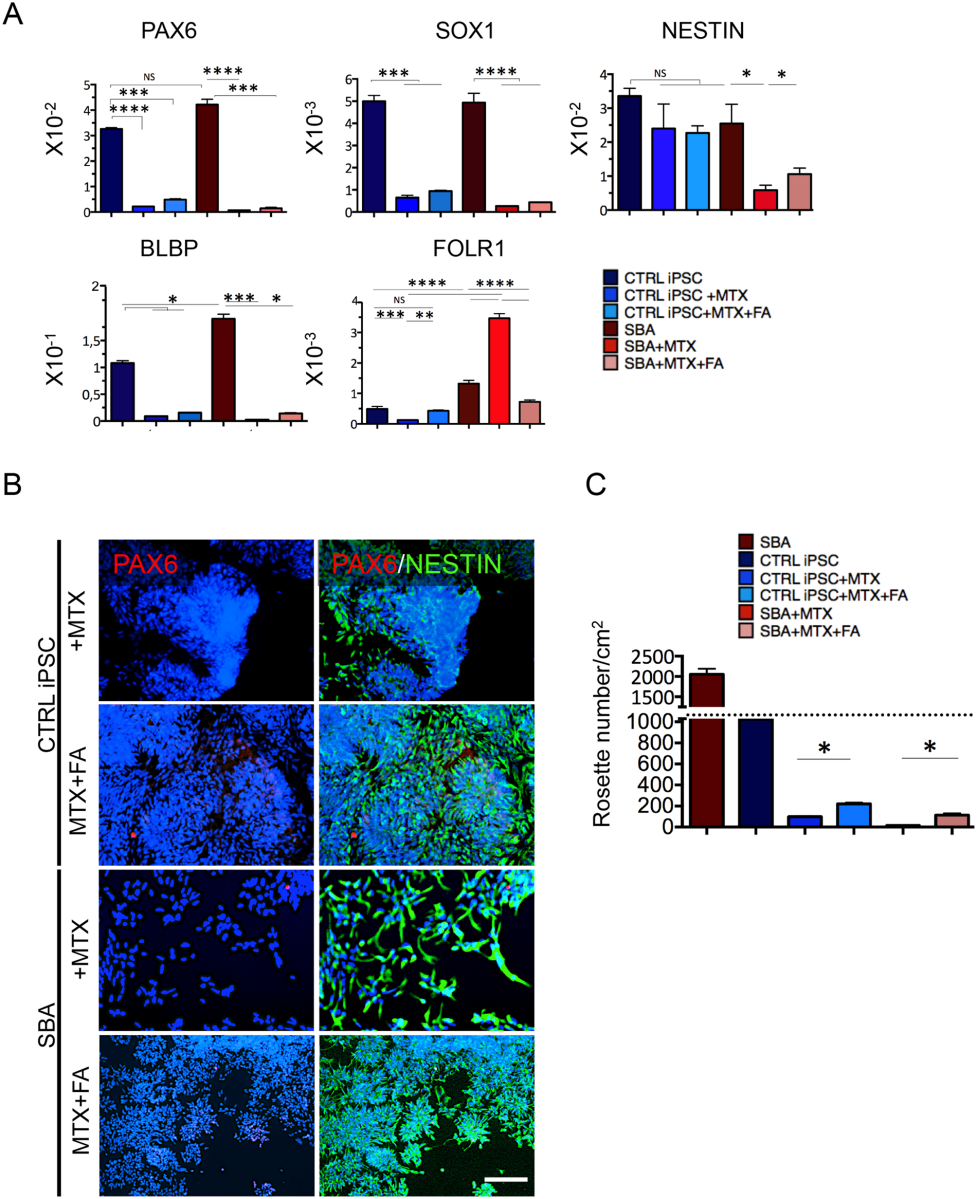
Effect of MTX exposure on SBA iPSC-derived NSCs and rosettes. (**A**) qRT-PCR data of CTRL iPSC and SBA-derived NSCs for early neural (*PAX6*, *SOX1, NESTIN*), RG (*BLBP*) and folate receptor 1 (*FOLR1*) markers at day 12 of neural differentiation is shown as relative expression to housekeeping gene *GAPDH*. NS, not significant. Data are plotted as average ± SEM. ****P < 0.0001 CTRL iPSC *vs* CTRL iPSC + MTX and SBA *vs* SBA + MTX, ***P < 0.001 CTRL iPSC *vs* CTRL iPSC + MTX/FA and SBA *vs* SBA + MTX/FA (*PAX6*), ***P < 0.001 CTRL iPSC *vs* CTRL iPSC + MTX and CTRL iPSC *vs* CTRL iPSC + MTX/FA, ****P < 0.0001 SBA *vs* SBA + FA and SBA *vs* SBA + MTX/FA (*SOX1*), *P < 0.05 SBA *vs* SBA + MTX, SBA vs SBA + MTX/FA and SBA + MTX *vs* SBA + MTX/FA (*NESTIN*), *P < 0.05 CTRL iPSC *vs* CTRL iPSC + MTX, CTRL iPSC *vs* CTRL iPSC + MTX/FA, CTRL iPSC *vs* SBA, SBA *vs* SBA + MTX/FA, ***P < 0.001 SBA *vs* SBA + MTX (*BLBP*), ****P < 0.0001 CTRL iPSC *vs* SBA, CTRL iPSC + MTX *vs* SBA + MTX, CTRL iPSC + MTX *vs* SBA + MTX/FA, SBA *vs* SBA + MTX, SBA + MTX *vs* SBA + MTX/FA, ***P < 0.001 CTRL iPSC *vs* CTRL iPSC + MTX and SBA *vs* SBA + MTX/FA(*FOLR1*). N = 4 independent experiments per iPSC line. (**B**) IF staining for early neural markers PAX6 (red) and NESTIN (green) in CTRL iPSC and SBA-derived neural rosettes, at day 18 of neural differentiation. Representative images of CTRL1#2 and SBA2#1 are shown. Nuclei were counterstained with DAPI (blue). Scale bar = 100 μm. (**C**) Quantification of CTRL iPSC and SBA-derived neural rosettes number, at day 18 of neural differentiation. Data are plotted as average ± SEM. *P < 0.05 CTRL iPSC + MTX *vs* CTRL iPSC + MTX/FA, SBA + MTX *vs* SBA + MTX/FA. N = 4 independent experiment per iPSC line.

qRT PCR analysis at day 12 from neural induction showed that MTX treatment significantly downregulated *PAX6, SOX1* and *BLBP* in SBA and control lines (Fig. 5A). Interestingly, *NESTIN* was significantly downregulated only in SBA lines, differently from the control lines where MTX treatment did not impose any effect on *NESTIN* expression. The combined exposure of MTX + FA resulted in a significant upregulation of *NESTIN* in SBA lines. Surprisingly, MTX treatment caused upregulation of *FOLR1* in SBA lines *(p* < *0.001)*, different from the controls, where *FOLR1* was downregulated upon MTX exposure. MTX + FA combined administration resulted in a significant downregulation of *FOLR1* in SBA lines *(p* < *0.0001)* and upregulation in control lines.

IF analysis at day 18 of neural differentiation showed that MTX treatment dramatically affected the rosette formation in SBA and control lines. All the lines were positive for NESTIN and negative for PAX6 staining (Fig. 5B). While control lines were still presenting several immature rosettes, in SBA lines rosettes were totally absent. MTX + FA administration resulted appearances of immature rosettes in SBA and mature neural rosettes in control lines. Quantification of the amount of neural rosettes confirmed that MTX exposure significantly affected the number in all cell lines, more severely in SBA lines (p < 0.0001) slightly rescued by FA exposure (Fig. 5C).

Altogether, MTX dramatically affected NSCs and neural rosette formation, more substantially in SBA lines and FA exposure partially rescued this severe phenotype in all cell lines. This phenomenon was more manifested in SBA lines, likely due to the MTX-induced increase of *FOLR1* gene differently from the controls.

### Limited chondrogenesis by SBA fetal iPSC lines and rescue of *ACAN* gene expression by FA administration

To evaluate the chondrogenic potential of SBA iPSCs and explore FA effects (4.5 μM) on cartilage formation, we induced chondrogenesis using a high-density micromass culture protocol^37^.

**Figure 6:**
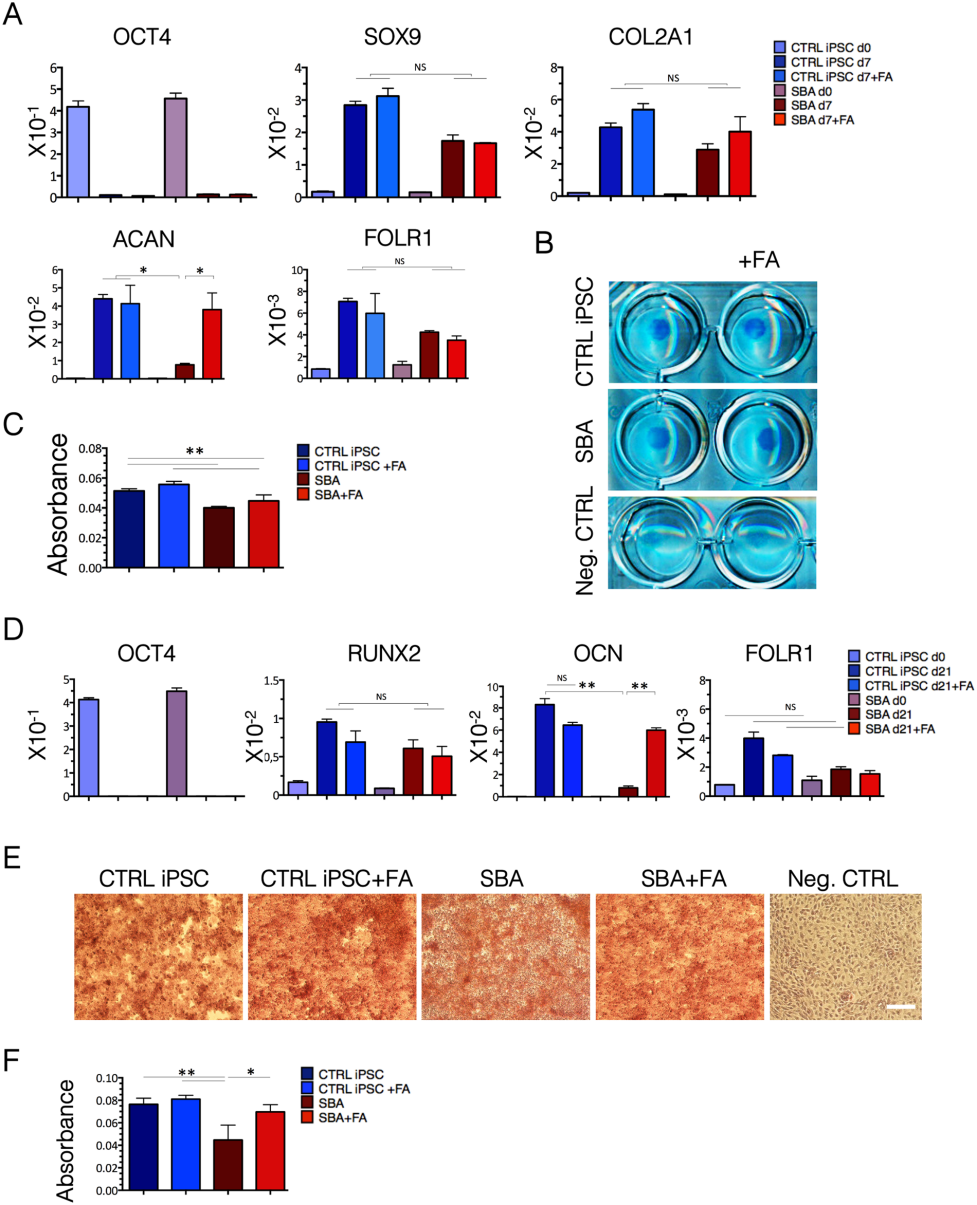
Osteo-chondrogenic commitment of SBA iPSC lines. (**A**) qRT-PCR analysis for pluripotency (*OCT4)*, chondrogenic (*SOX9, COL2A1, ACAN*) and folate receptor 1 (*FOLR1)* genes at day 0 and day 7 of chondrogenic induction is shown as relative expression to housekeeping gene *GAPDH* in CTRL iPSCs and SBA-derived chondrocytes. NS, not significant. Data are plotted as average ± SEM. *P < 0.05 CTRL iPSC d7 *vs* SBA d7, CTRL iPSC + FA d7 *vs* SBA d7, SBA d7 *vs* SBA + FA d7 (*ACAN*). N = 4 independent experiments per iPSC line. (**B**) ACB staining of micromass cultured from CTRL iPSC and SBA-derived chondrocytes and negative CTRL (CTRL iPSC without chondrogenic induction) at day 7 of chondrogenic differentiation. Representative images of CTRL1#2 and SBA2#1 are shown. Scale bar = 1 mm. (**C**) Quantification of CTRL iPSC and SBA-derived chondrocytes at day 7 of chondrogenic differentiation, measured by ICN Titertek Multiskan Plus. Data are plotted as average ± SEM. **P < 0.01 CTRL iPSC *vs* SBA, CTRL iPSC *vs* SBA + FA, CTRL iPSC + FA *vs* SBA + FA. N = 4 independent experiments per iPSC line. (**D**) qRT-PCR data of CTRL iPSCs and SBA-derived osteoblasts for pluripotency (*OCT4)*, osteogenic (*RUNX2, OCN*) and folate receptor 1 (*FOLR1)* genes at day 0 and day 21 of osteogenic differentiation is shown as relative expression to housekeeping gene *GAPDH*. NS, not significant. Data are plotted as average ± SEM. **P < 0.01 CTRL iPSC d21 *vs* SBA d21, SBA d21 *vs* SBA + FA d21 (*OCN*). N = 4 independent experiments per iPSC line. (**E**) ARS staining of CTRL iPSC and SBA-derived osteocytes and negative CTRL (CTRL iPSC without osteogenic induction) at day 21 of osteogenic differentiation. Representative images of CTRL1#2 and SBA2#1 are shown. Scale bar = 100 μm. (**F**) Quantification of CTRL iPSC and SBA-derived osteoblasts at day 21 of osteogenic differentiation, measured by ICN Titertek Multiskan Plus. Data are plotted as average ± SEM. **P < 0.01 CTRL iPSC *vs* SBA, CTRL iPSC + FA *vs* SBA, *P < 0.05 SBA *vs* SBA + FA. N = 4 independent experiment per iPSC line.

At day 7 of chondrogenic differentiation, *OCT4* was downregulated in concomitance with upregulation of the chondrogenic markers (*SOX9, COL2A1* and *ACAN)* (Fig. 6A). *ACAN*, a rather late chondrogenic marker, was expressed at lower levels in SBA lines compared to the controls, and FA exposure resulted in its upregulation, reaching values similar to that in controls. In parallel, Alcian Blue (ACB) staining and Guanidine hydrochloride (GH) extraction were performed on day 7 cultures to assess glycosaminoglycan (GAG) deposition. The presence of extracellular matrix was confirmed by ACB staining in all FA treated/untreated cells (Fig. 6B), however, quantification of GH showed that SBA iPSCs presented significantly lower staining as compared to controls. FA treatment did not improve this defect in extracellular matrix production (Fig. 6C).

Taken together, our results suggest that SBA iPSCs had a reduced chondrogenic potential compared to the controls, and FA administration improved *ACAN* expression but not cumulative GAGs deposition.

### Limited osteogenesis by SBA fetal iPSCs and rescue by FA administration

To evaluate the effect of FA treatment (4.5 μM) on osteogenic lineage commitment of SBA iPSCs, we differentiated SBA iPSCs into osteoblasts in the presence and in the absence of FA^38^. Runt-related transcription factor 2 (*RUNX2)* and the bone formation marker osteocalcin (*OCN)* were upregulated in all samples in concomitance with *OCT4* down regulation at day 21 of osteogenic induction. However, the expression of *OCN* was significantly lower in SBA lines compared to controls. Interestingly, FA treatment rescued the *OCN* expression in SBA lines (Fig. 6D). Mineral deposition assessed by Alizarin Red S (ARS) staining showed that most cells differentiated toward osteoblasts (Fig. 6E). Nevertheless, quantification of ARS staining clearly demonstrated that SBA cultures contained less mineral deposition compared to control cell lines. Again, upon FA treatment mineral deposits were increased in SBA lines similar to controls, where FA administration maintained homogeneous mineral deposition (Fig. 6F).

These results show that SBA iPSCs had lower osteogenic potential compared to controls and FA treatment significantly improved osteogenic differentiation ability in SBA cell derivatives.

### Downregulation of *PAX7* and upregulation of *PAX3* gene upon FA exposure in SBA fetal iPSCs subjected to myogenic differentiation

**Figure 7:**
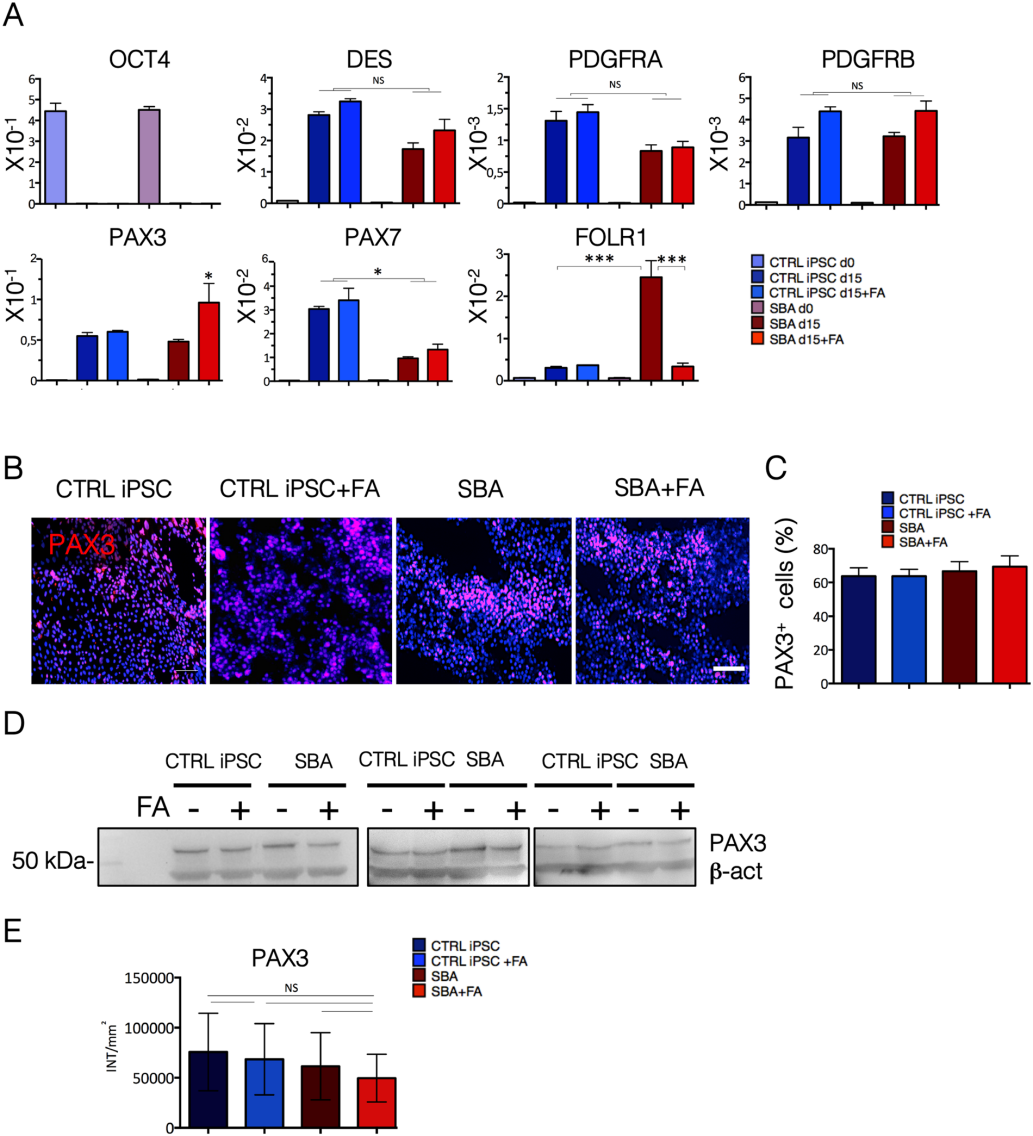
Early myogenic commitment of SBA iPSC lines. (**A**) qRT-PCR data of CTRL iPSC and SBA lines for pluripotency (*OCT4)*, mesodermal (*DES, PDGFRA, PDGFRB*), early myogenic (*PAX3, PAX7*) and folate receptor 1 (*FOLR1)* genes at day 0 and day 15 of myogenic differentiation presented as relative expression to housekeeping gene *GAPDH*. NS, not significant. Data are plotted as average ± SEM. *P < 0.05 SBA d15 *vs* SBA d15 + FA, CTRL iPSC d15 *vs* SBA d15 + FA and CTRL iPSC d15 + FA *vs* SBA d15 + FA (*PAX3*), *P < 0.05 CTRL iPSC d15 *vs* SBA d15, CTRL iPSC + FA d15 *vs* SBA + FA d15 (*PAX7*), ***P < 0.001 CTRL iPSC d15 *vs* SBA d15, SBA d15 *vs* SBA + FA d15 (*FOLR1*). N = 4 independent experiment per iPSC line. (**B**) IF staining for PAX3 (red) in CTRL iPSCs and SBA-derived myogenic progenitors at day 15 of myogenic induction. Representative images of CTRL1#2 and SBA2#1 are shown. Nuclei were counterstained with DAPI (blue). Scale bar = 100 μm. (**C**) Enumeration of PAX3 + cells in CTRL iPSC and SBA lines at day 15 of myogenic induction shown as percentage. Data are plotted as average ± SEM. N = 4 independent experiment per line. (**D**) WB analysis for PAX3 protein (53 kDa) in CTRL iPSCs and-derived myogenic progenitors. Housekeeping protein β Actin (45 kDA) used as an internal control. The images were cropped on the black lines. Full images of gels/blots can be found in Supplementary Fig. S7. (**E**) Enumeration of PAX3 protein using QuantityOne Software. NS, not significant. N = 3 independent experiments per line.

To evaluate FA exposure effects on early myogenic commitment of SBA iPSCs, we induced early myogenic induction in the presence and in the absence of FA (4.5 μM), based on mesodermal induction and skeletal muscle differentiation approach. At day 15 from myogenic induction, down regulation of *OCT4* followed by upregulation of mesodermal and myogenic markers was observed (Fig. 7A). Expression of mesodermal markers *DESMIN, PDGFRA* and *PDGFRB* was similar in all cell lines and was not affected by FA exposure. The early myogenic transcription factor *PAX7* was significantly downregulated in SBA lines and FA exposure did not show any effect on its expression. In addition, *FOLR1* was significantly upregulated in SBA lines in untreated conditions and downregulated upon FA treatment as observed previously for neural induction. We noticed that SBA lines also significantly upregulated the early myogenic transcription factor *PAX3* upon FA treatment. However, further evaluation of PAX3 protein by IF and WB showed similar protein content in myogenic progenitors derived from SBA and control lines even in the presence of FA (Fig. 7B–E).

In summary, our results suggest that early myogenesis of SBA iPSCs was not dramatically affected, although *PAX7* was downregulated, and FA administration induced an upregulation of *PAX3* transcription factor.

## Discussion

NTDs are severe congenital anomalies affecting 1 in every 1,000 pregnancies. FA supplementation is able to prevent occurrence by up to 70%, however the mechanisms behind the action of FA are not clear yet. Given the fact that animal models cannot completely recapitulate human NTDs and clinical phenotype, the needs of additional models, including human *in vitro* models were urgent. Hence, for the first time, we introduced a human cell model derived from SBA fetuses, one of the most severe forms of NTDs. We used different cell sources derived from SBA fetuses, including FSFs and AFSCs, to generate human iPSCs and employed a transgene-free SeV reprogramming approach to avoid integration into the host genome^39,40^. Previous studies showed that human iPSC-derived neural crest stem cells integrated and differentiated into neural lineage when transplanted in large animal models of NTD^41^. Hence human SBA iPSC derivatives could, in principle, be *in vitro* manipulated and tested *in vivo* for their regenerative potential.

Neural tube formation and closure are complex processes and may be represented *in vitro* by NSC generation and rosette formation. As a consequence, SBA iPSC-derived neural rosettes may reveal some morphological and gene signature abnormalities. To our surprise, NSCs generated from SBA lines formed self-organized structures and expressed set of early neural genes (*PAX6, SOX1, NESTIN*) similar to the control cell lines. Furthermore, they were correctly polarized, as evidenced by a proper localization of N-CAD and ZO-1 proteins surrounding the lumen already at day 18 of neural induction. Despite these similarities, a number of defects were noted. For example SBA rosettes showed increased expression of the *BLBP* gene, an early RG marker, suggesting altered RG commitment. BLBP plays a role in the establishment of the RG fibres in the developing brain, and impaired RG commitment can alter neural migration and lead to abnormal CNS development^42,43^. Additionally, previous studies showed that BLBP is highly expressed in different types of tumours^44–46^, including neuroblastoma, one of the most common neurogenic tumours of infancy. In view of this, SBA lines would provide a novel platform to further explore the common characteristics with neuroblastoma.

Our results showed that neural rosettes derived from SBA lines were larger in number, yet significantly smaller in size. FA exposure corrected the aberrant phenotype of neural rosettes. This confirms that indeed, appropriate dose of FA is necessary to guarantee the proper formation of neural rosettes. It is still true; that basal culture medium contained few micromoles of FA, and all cell lines, including not treated, received a few amount of FA. However, our analysis demonstrated that extra amount of FA positively impacted on SBA neural rosettes formation. Indeed, some foods are rich with folate (naturally or by fortification), but still additional 400 micrograms (in some cases even more) per day FA supplementation is recommended during the first trimester of pregnancy, which is the crucial period of neural tube formation, closure and the CNS maturation.

NTDs occur at early stage of embryonic development, when NSCs are still actively proliferating. Our data showed that at day 6 of neural induction NSCs from SBA lines were proliferating similar to control lines, and FA exposure increased the amount of Ki67+ cells in pathologic and healthy lines. On the contrary, at day 18 of neural induction neural rosettes derived from SBA lines showed a serious reduction in number of Ki67+ cells. Previous studies reported that disrupted NSC proliferation causes several diseases like autism or schizophrenia^47–49^. Further experiments are necessary to precisely define the molecular mechanism behind the NSC proliferation impairment within neural rosettes. Additionally, it has previously been shown that FA influenced proliferation and maturation of fetal neural tube cells from Splotch mutant mouse^32^. Consistent with these reports, FA administration in our human cell model greatly improved the number of Ki67+ cells within neural rosettes that became larger and similar to controls.

To better mimic neural tube maturation process, we generated 3D spheroids from neural rosettes. Our results demonstrated that also 3D spheroids derived from SBA neural rosettes were significantly smaller and presented less Ki67+ cells compared to the controls. Consistent to our results, *Lancaster et al*. showed that cerebral organoids derived from neurodevelopmental disorder microcephaly patient-derived iPSCs resembled small brain structures too^50^. Our data highlighted that the size and amount of early neural tube structures can be crucial too for CNS development and, eventually, for the neural tube closure. FA exposure greatly rescued the size of 3D neural tube structures and the number of Ki67+ cells. Notably, Ki67+ cell fraction was increased mainly in the midline of the lumen, where ZO-1 was expressed too. Polarization, adherence junction interactions are crucial for neural tube closure^51^ and tissue remodelling^52^. Our data suggest that additional amount of FA influenced the morphogenetic processes of neural tube by increasing proliferation and the maturation of the luminal structures. We also evaluated early neural differentiation potential of 3D spheroids, to check upon the hypothesis that the decreased proliferation was likely to be due to the increased number of the early-generated neurons in SBA lines. Our findings demonstrated that β-III Tubulin+ cells were less frequent in SBA derived spheroids as compared to the controls. FA greatly increased the fraction of β-III Tubulin+ cells in SBA and control lines, indicating the important role of FA for early neural differentiation process too. Consistent to our results previous studies also demonstrated the positive influence of FA on fetal neural stem cells differentiation from NTD mouse model^32^.

We next assessed NSC differentiation and neural rosette formation upon folate metabolism disruption through acute treatment with folate antagonist MTX. Indeed, our data demonstrated that interference with the folate metabolism resulted in down regulation of early neural markers that dramatically affected neural rosette formation in both SBA and control lines. In agreement with this, previous studies showed that absence of FA in neural differentiation medium also significantly inhibited NSCs proliferation and the expression of neural stem cell markers^3^. In our previous study we demonstrated that MTX negatively regulated neurogenesis also in human AFSC culture^53^. It has been also shown that folate deficiency or MTX treatment affected proliferation of embryonic murine NSCs causing NTDs^54^. Additionally, it has been demonstrated that FA administration rescued *de novo* proliferation and neurosphere formation in mouse primary cultured NSCs exposed to MTX^55^. This is in line with our observations in human cell model that FA supplementation impacted positively on neural gene expression profile and rosette formation, disrupted by MTX exposure. Thus our data support a mechanistic basis for clinical recommendations of FA administration during MTX treatments. Intriguingly, *FOLR1* was positively regulated by extracellular folate depletion, but upon MTX treatment upregulation was only seen in SBA-derived NSCs. This suggests that SBA-derived NSCs were extremely sensitive to folate depletion and promptly induced *FOLR1* activation in an attempt to increase folate signalling. Indeed, MTX + FA treatment resulted in significant downregulation of *FOLR1* in SBA lines. The upregulation of *FOLR1* observed in specific malignant tumours of epithelial origin^56,57^ supports the idea that SBA-derived NSCs display a more immature phenotype compared to healthy NSCs.

Since previous reports showed impairments in mesodermal tissue formation in both mouse and human NTDs, we differentiated SBA iPSC lines toward the three major mesodermal lineages: chondrogenic, osteogenic and myogenic cells. Our data demonstrated that osteogenic and chondrogenic differentiations were less efficient in SBA lines compared to control lines. FA exposure significantly increased in SBA lines the expression of the cartilage matrix marker *ACAN* essential for cartilage formation during development^58^, however, did not affect the overall GAG deposition, likely because of the short term of FA exposure. FA significantly increased also the osteogenic marker *OCN* and mineral deposition in SBA lines in appropriate culture regiments. In the line to our results, previous studies also demonstrated reduced expression of cartilage matrix genes *COL2A1* and *ACAN* and limited chondrogenesis in an iPSC-based model from patients affected by the congenital disorder, achondroplasia^59^. Furthermore, previous studies showed that dietary supplementation of FA rescued cartilage defects in transgenic mice and that developing chondrocytes required folate for proliferation and/or differentiation^60^.

Finally, our study showed that early myogenesis was not impaired in SBA iPSCs, although *PAX7* was significantly downregulated and *PAX3* was upregulated upon FA treatment in SBA lines compared to controls. These findings are particularly relevant since *PAX3* and *PAX7* are essential for normal embryonic development and are specifically expressed in the neural tube and the developing somite^61^. The Splotch (*Pax3-null*) mutant mice exhibit 100% of penetration of neural tube and loss-of-function mutations of *PAX3* are linked to Waardenburg syndrome and NTDs in humans^62^. Our previous study also showed overexpression of *PAX3* in AFMSCs isolated from the SBA lamb model^63^ thus emphasising the importance of this gene in the pathogenesis of NTD.

Future experiments on our SBA model should be dedicated to find out the possible mechanisms behind the disease phenotypes and FA exposure effect. However, it is still noteworthy to show for the first time how proper amount of FA positively impacted on several phenotypes represented by SBA human iPSC-derived model. Moreover, several signalling pathways contribute to neural tube formation and could be altered in our SBA model. For instance, recent studies showed that γ-secretase inhibitors, preventing Notch signalling, affected *PAX6*+ and *Ki67*+ cells in human iPSCs-derived rosettes^64^. In support of this are other studies demonstrating that FA greatly stimulates Notch signalling and cell proliferation in embryonic NSCs^65^. Alteration of Notch signalling could be another explanation for altered rosette phenotype and be directly linked to Ki67+ cell depletion. However, it is likely not sufficient to consider only Notch signalling to be implicated in the beneficial effect of FA administration that we observed. Indeed, more than 5,000 genes responded to FA exposure in NSCs generated from Rhesus monkey ES cells. These are mainly involved in cell division, morphogenesis, cell migration and metabolism as recently reported^3^.

In conclusion, we have set up the first human SBA cell model that clearly recapitulates defective early neurogenesis. We believe our human SBA cell model can be used for neurodevelopmental studies and can be further explored to reveal novel mechanistic insights into the pathogenesis of NTDs, for drug screenings purposes and *in vivo* regenerative studies.

## Methods

### Samples collection

Written informed consent to collect samples was obtained from all participants and the study was approved by the Human Research Ethics Committee of IRCCS University Hospital San Matteo Foundation and the Ethics Committee of the University Hospitals Leuven (ML9167). In addition to this, all methods were performed in accordance with the relevant guidelines and regulations. For the generation of SBA lines skin tissues from 3 SBA fetuses (type-myelomeningocele) and 1 amniotic fluid sample (from pregnant patient of SBA fetus (type-myelomeningocele) were taken between 21–25 gestational weeks, during fetoscopic intervention and amniocentesis at IRCCS University Hospital San Matteo Foundation and at the University Hospitals Leuven. For control lines we used skin tissues from 2 healthy adult donors and 1 healthy amniotic fluid sample (gestational week 23) from healthy fetus.

### Isolation of fibroblasts from skin tissue

Skin tissues from SBA and healthy donors were seeded in cell culture plates (Nunc^TM^), coated with collagen (Sigma), in the presence of fibroblast growth medium (FGM), consisting of KO-DMEM (Invitrogen), 2 mM L-glutamine (Invitrogen), 10% fetal bovine serum (FBS; Invitrogen), 100 U ml^−1^ penicillin and 100 mg ml^−1^ streptomycin (Invitrogen). Cultures were monitored daily and medium was carefully refreshed every 3 days. Around day 10 tissues were removed from culture, cells were washed twice with Phosphate-buffered saline (PBS; Thermo Fisher) and detached using TrypLE (Invitrogen). Cells were collected and centrifuged at 1,200 for 5 minute and expended using collagen coating.

### Isolation of stem cells from amniotic fluid sample

In our previous study we isolated and deeply characterized SBA and healthy-derived AFSCs, showing that both lines exhibited mesenchymal stem cells characteristics^53^. Briefly, amniotic fluid sample was transferred in cell culture tube and centrifuged at 1,200 for 10 minute. Cell pallet resuspended in mesenchymal basal medium (DMEM, 20% FBS, 100 U ml^−1^ penicillin, 100 mg ml^−1^ streptomycin and 2 mM L-glutamine) and incubated at 37 °C with 5% humidified CO_2_. After 48 h, nonadherent cells were removed by washing the plate with PBS, and the medium was replaced to FGM described above. Upon reaching 75% confluence, cells were detached using TrypLE and seeded at a split ratio of 1:4. Cells were daily monitored, and expended.

### Generation of iPSCs using non-integrating SeV reprogramming approach

iPSC generation was performed using SeV Reprogramming Kit, according to the manufacturer’s instructions (CytoTune-iPS 2.0 Sendai Reprogramming Kit, Invitrogen). Fetal, healthy skin fibroblasts and amniotic fluid derived stem cells were seeded at 20.000 cells/cm^2^ and transduced with Sendai viruses encoding for OSKM *(OCT3/4, SOX2, KLF4* and *c-MYC)* factors. Briefly, after three-four passages cells where seeded in 6-well plates in FGM, consisting of KO-DMEM supplemented with 10% FBS, 1 mM L-glutamine, 50 U ml^−1^ penicillin and 50 mg ml^−1^ streptomycin. When cells reached 70–80% confluence, they were infected with SeV. After 24 h medium was refreshed. Cells were daily monitored, and at day 7 cells were plated on inactivated mouse embryonic fibroblasts (iMEFs) feeder layer in 10 cm culture dishes. The following day medium was switched to iMEF-conditioned hPSC medium, consisting of DMEM/F-12 GlutaMAX (Invitrogen) supplemented with 20% Knockout serum (KSR) (Invitrogen), 10 ng ml^−1^ FGF2 (PeproTech), 100 μM non-essential amino acids (NEAA; Life Technologies), 100 μM 2-mercaptoethanol (Invitrogen), 50 U ml^−1^ penicillin and 50 mg ml^−1^ streptomycin with daily refreshment.

First iPSC-like colonies appeared already after 2 weeks. By day 20–23 of culture, iPSC colonies became morphologically similar to human ESCs. Several colonies from each clone were manually picked and expanded on iMEF feeder layer until passage 8–10. Subsequently, colonies were transitioned to feeder-free culture conditions, using qualified Matrigel (qM, Corning)-coating and were grown in mTeSR1 media (Stem Cell Technologies). iPSC colonies were expanded manually or by enzymatic treatment using Dispase I protease (Sigma Aldrich) using qM-coating and mTeSR1 media.

### Embryoid body formation from human iPSCs

Human iPSC colonies were grown on feeder free conditions, using Geltrex (Life Technologies) coating, in Essential 8 medium (Thermo Fisher). After 4–5 days iPSC colonies were dissociated by enzymatic treatment using EDTA Accutase (Sigma Aldrich) for 3 minutes at 37 °C. Cells were carefully resuspended few times in Essential 6 medium (Thermo Fisher) and transferred in TC-Treated 24-Well Plates (Corning Costar). Medium was carefully refreshed every 2 days. At day seven embryoid bodies were collected and processed for further analysis.

### Neural induction and neural rosettes formation: culture conditions

Neural induction was performed using well established protocol^66^ with a few modifications. Briefly, human PSCs were counted and 500, 000 cells from each line were cultured in feeder- free conditions using qM in mTeSR medium. 10 µM Y-27632 dihydrochloride (ROCK-I, Tocris Bioscience) was added to the medium during plating the cells. Medium was refreshed every day. At day 4–5 cells were detached by enzymatic treatment using Accutase, counted and 2.000 000 cells from each line were again plated using the same culture condition. Next day, when all the cells reached 100% confluence, medium was changed to neural induction (described below) medium. Cells were maintained in neural induction medium for 12 days and medium was refreshed every day. At day 12, cells were incubated with Dispase I protease (Life Technologies) for 5 minute at 37 °C, and gently washed with neural maintenance medium (described below) three times. The clumps then plated on Laminin (Sigma Aldrich)-coated plates in neural induction medium. Next day, medium was replaced with neural maintenance medium and 20 ng/mL FGF2 (PeproTech) was added daily for the first four days. After 4 days, cells were maintained in neural maintenance medium and medium was refreshed every other day.

Neural maintenance medium: 1:1 mixture of B27 and N2 containing medium. B27 medium consisted of Neurobasal (Gibco), 1× B-27 Supplement (Gibco), 200 mM L-glutamine, 50 U ml^−1^ penicillin and 50 mg/ml^−1^ streptomycin. N-2 medium consisted of DMEM/F-12 GlutaMAX (Gibco), 1× N-2 Supplement (Gibco), 5 μg ml^−1^ human insulin solution (Sigma Aldrich), 1 mM l-glutamine, 100 μm NEAA, 100 μM 2-mercaptoethanol, 50 U ml^−1^ penicillin and 50 mg ml^−1^ streptomycin.

Neural induction medium: Neural maintenance medium supplemented with 500 ng/mL^−1^ recombinant human Noggin (PeproTech) and 10 μM SB431542 (Tocris Bioscience).

### Generation of 3D neural tube like spheroids and embedding in paraffin

At day 18 of neural differentiation several rosettes were manually isolated from culture plates and were grown as spheroids in V-shape, 96-well culture plates (Nun clon^TM^ Delta surface) in neural maintenance medium. Medium was carefully refreshed every 3 days. Spheroids were maintained in this condition for 7 days. At day 7 spheroids were collected in 1.5 ml cell culture tubes and fixed with 4% PFD at 4 °C, overnight. The day after, spheroids were subsequently washed in PBS and stored at 4 °C in 70% ethanol solution, encapsulated in 2% agarose gel and stored at 4 °C in 70% ethanol solution. Finally, encapsulated spheroids were processed using the Excelsior tissue processor and embedded in paraffin using the HistoStar (Thermo Scientific).

### Chondrogenic differentiation: culture conditions

Undifferentiated iPSCs were cultured on iMEFs feeder in iMEF-conditioned hPSC medium described above. Cells were dissociated by enzymatic treatment using Accutase incubation for 5 minute at 37 °C. Dissociated cells then plated in 10 µL spots at high density. Plates were incubated for 2 hours to allow attachment of the cells. After 2 hour, maintenance medium (DMEM-F12, 10% FBS and 10% KSR) was added. Next day, the maintenance medium was changed to incomplete chondrogenic medium, consisting of DMEM-F12, supplemented with 10 μg ml^−1^ Insulin-transferrin-selenium (ITS; Sigma Aldrich), 40 mg/mL L-proline (Sigma Aldrich), 0.1 mM NEAA, 50 μM L-ascorbic acid-2-phosphate (Sigma Aldrich) and 100 μM Dexamethasone (Sigma Aldrich). After additional 24 h complete chondrogenic medium was added (incomplete medium supplemented with 100 ng/mL BMP2 (PeproTech) and 10 ng/mL TGF-β1 (PeproTech)). Medium was changed every 2 days. At day 7, experiments were stopped and molecular characterizations carried out.

### Osteogenic differentiation: culture conditions

Undifferentiated iPSCs were cultured on iMEFs feeder in iMEF-conditioned hPSC medium described above. Cells were incubated with Collagenase IV (Sigma Aldrich) for 5 minute at 37 °C. Cells were then washed with culture medium 3 times, and the colonies were broken using a cell scraper (Corning). Cells were grown in ultralow attachment plates (Corning) as aggregates in mesodermal induction medium consisting of IMDM (Gibco) supplemented with 20% FBS, 0.1 mM NEAA, 2 mM L-glutamine, 50 U ml^−1^ penicillin and 50 mg ml^−1^ streptomycin, 4.5 mM 1-Thioglycerol (Sigma Aldrich), 50 μg/ml L-ascorbic acid-2-phosphate and 200 μg/mL holo-Transferrin human (Sigma Aldrich). Medium was carefully refreshed every 2 days. At day 5, aggregates were replated in Gelatine (Sigma Aldrich) coated plates in osteogenic induction medium, consisting of Mem Alpha (Gibco) supplemented with 10% FBS, 100 nM Dexamethasone, 10 mM β-Glycerophosphate disodium salt hydrate (Sigma), 50 μM L-ascorbic acid-2-phosphate, 2 mM L-glutamine and 50 U ml^−1^ penicillin and 50 mg ml^−1^ streptomycin. Medium was changed every 2 days. At day 21 of osteogenic differentiation, experiments were stopped and molecular characterizations carried out.

### Mesodermal induction and early myogenic differentiation: culture conditions

Undifferentiated iPSCs were cultured on iMEFs feeder in iMEF-conditioned hPSC medium described above. Cells were incubated with Collagenase IV for 5 minute at 37 °C. Cells were then washed with culture medium 3 times, and the colonies were broken using a cell scraper. Cells were grown in ultralow attachment plates as aggregates in mTeSR medium for 3 days. At day 3 culture medium was changed to myogenic differentiation medium, consisting of IMDM, 15% FBS, 10% horse serum (Sigma Aldrich), 1% chicken embryo extract (Seralab), 50 μg/mL L-ascorbic acid-2-phosphate and 4.5 mM 1-Thioglycerol. Medium was carefully refreshed every 2 days. At day 9, aggregates were replated in gelatine-coated plates in the same medium. Medium was refreshed every 3 days. At day 16, experiments were stopped and molecular characterizations carried out.

### Cell viability assay

Cell pallet was resespendent in 1000 μL culture medium, from which, 10 μL aliquot was resuspended in 10 μL Trypan Blue (Life Technologies) and counted in automated cell counter (Invitrogen). The exact number of live/dead cells and viability were evaluated.

### Human iPSC ScoreCard assay

hPSC ScoreCard assays (Life Technologies) were performed according to manufacturer’s instructions on a Viia7 RT-PCR System (Applied Biosystems) using the hpsc-ScoreCardviia-7-384-wells template. Briefly, cDNAs from each sample were added to TaqMan® Master Mix and transferred to the 384-well plate with a multichannel pipette. Gene expression data set was analysed using cloud-based analysis software (Life Technologies).

### Teratoma formation assay

All the mouse experiments were carried out in accordance with European guidelines on animal research and approved by the ethics committee at the University of Leuven.

Human iPSCs were collected by enzymatic dissociation using Accutase and 5 × 10^5^ cells were resuspended in 100 µL PBS and injected with 100 µL qM subcutaneously in the back of immunodeficient RAG2−/− γc−/− 5–6 week-old mice. In 5–8 weeks mice were sacrificed and dissection was made to obtain teratomas. Teratomas were then fixed in 4% Paraformaldehyde (PFD; Sigma Aldrich) overnight. Next day teratomas were washed in 70% Ethanol (Sigma Aldrich) then embedded in paraffin. After sectioning, the presence of cells from the three germ layers was assessed following haematoxylin and eosin staining (H&E). Additionally, frozen sections embedded in Optimal Cutting Temperature (OCT) matrix were processed for IF analysis.

### RNA extraction and quantitative real-time PCR (RT-qPCR)

The GeneElute Mammalian Total RNA Miniprep Kit (Sigma) was used to extract RNAs, following the manufacturer’s protocol. Briefly, 1 μg of RNAs was retrotranscribed using the SuperScriptTM III First-Strand Synthesis SuperMix Kit (Invitrogen) and diluted in DEPC water. Primers (Supplementary Table S1) for RT-qPCR were designed using Primer 3 (http://frodo.wi.mit.edu/primer3/) and tested for their efficiency. RT-qPCR was performed, according to the SYBR Green Mix (Invitrogen) protocol, on real-time system Realplex2 Master Cycler (Eppendorf) or on ViiA7 Real-Time PCR system (Invitrogen).

### Immunofluorescence (IF) analysis

Cells were fixed with 4% PFD for 10–20 minute at room temperature (RT), then permeabilized in Triton-X-100 (Sigma Aldrich) at RT for 30 minute. Cells were washed and blocked in 10% donkey serum (Sigma Aldrich) for 30 minute. Cells were then incubated with primary antibodies (Supplementary Table S2) overnight at 4 °C followed by the appropriate secondary antibodies (1:500) incubation for 30 minute at RT. The nuclei were counterstained with 4′,6-diamidino-2-phenylindole (DAPI; Sigma Aldrich) for 3–4 min, and coverslips (Corning) were mounted on slides with FluorSave (Merck Millipore). Neural rosettes were not mounted by cover sleeps and in stead PBS was added in each well. Florescent imaging was obtained using Eclipse Ti microscope (Nikon), nuclei and rosette size measurements were performed using Image-Pro Plus 6.0 software. Quantification and measurement analyses were performed using Image-Pro Plus 6.0 software.

### Immunostaining of spheroids

Immunostaining performed on paraffin embedded spheroids sections. Breefly, after melting the paraffin and rehydrating, the sections were washed with PBS + 0.2% Triton-X-100. Then antigen retrieval (Dako Target Retrieval Solution (1x)) sections were permeabilised with 0.2% PBST for 5 minute, then blocked with 0.2% PBST + 5% donkey serum. Subsequently, slides stained with primary antibodies overnight at 4 °C. Next day, slides were washed with 1xPBST 3–4 times, afterwords incubated with secondary antibodies (1:500) for 30 minute at RT. The nuclei were stained using DAPI for 3–4 min. Slides were mounted with prolong Gold mounting medium (Thermo Fisher) and the signals were detected using Eclipse Ti microscope (Nikon).

### Alcian blue (ACB) staining and Guanidine hydrochloride (GH) extraction

Cells were fixed in ice-cold methanol for 1 h and kept at 4 °C for the following washes with PBS and MilliQ water. Subsequently, 1 ml of 0.1% ACB (Sigma) in 0.1 M HCl was added followed by 1 h incubation at RT. After incubation, the cells were washed with MilliQ and the plates were scanned and images were taken. For quantification, plates were incubated at RT, 300 µL 6 M GH (Merck) overnight with gentile agitation. The solution was then removed and 100 µL aliquots were transferred to a 96 well plate, diluted with 100 µL dH_2_0 and read at 620 nm by ICN Titertek Multiskan Plus.

### Alizarin Red S (ARS) staining and Cetylpyridinium chloride (CPC) extraction

At day 21 of osteogenic differentiation cells were fixed in ice-cold methanol (Sigma Aldrich) for 1 hour and incubated at 4 °C. Cells then were washed with PBS and MilliQ water (Sigma Aldrich). Subsequently, 1 mL of 1% ARS (Sigma Aldrich) solution (pH 4.1 diluted in water) was added to each well and incubated for 1 hour at RT. Cells were washed with MilliQ water until cleared of any dye. Then, ARS was distained for quantitative analyses. Briefly, cells were incubated with 300 µL 10% CPC (Sigma Aldrich), at RT overnight with gentle agitation. Next day the solution was removed and 100 µL aliquots were transferred to a 96 well plates, diluted in 100 µl dH_2_0 and read at 570 nm by ICN Titertek Multiskan Plus (Thermo Fisher).

### Protein extraction and Western blot analysis

At day 16 of myogenic induction cells were lysed in RIPA buffer (Sigma Aldrich) supplemented with 10 mM NaF, 0.5 mM Sodium Orthovanadate, 1:100 Protease Inhibitor Cocktail and 1 mM Phenylmethylsulfonyl Fluoride. Equal amount of protein were loaded in sample-loading buffer (50 mM Tris-HCl, pH 6.8, 100 mM DTT, 2% SDS, 0.1% bromophenol blue, and 10% glycerol) afterwords transferred in to nitrocellulose membranes. The membranes were blocked with Tris-buffered saline consisting of 0.05% Tween and 5% nonfat dry milk and incubated overnight at 4 °C with the primary antibody PAX3 (1:5000) and normalized for Beta-Actin (Millipore). After incubation with HRP-conjugated secondary antibody (1:5000, Invitrogen) for 1 hour at RT, specific bands were detected with Chemiluminescent Peroxidase Reagent (Sigma Aldrich) and pictures were taken with GelDoc system (Bio-Rad). Quantitation was performed using the QuantityOne Software.

### Statistics

Data were statistically analysed using Prism software (GraphPad Prism 6). The results were reported as the mean ± standard error of the mean (SEM). Differences between groups were examined for statistical significance using Student’s *t*-test or ANOVA. Significance was set at *P < 0.05, **P < 0.01 and ***P < 0.001, ****P < 0.0001.

## Electronic supplementary material

Supplementary Information
