# Supplementary material for: Folic Acid Exposure Rescues Spina Bifida Aperta Phenotypes in Human Induced Pluripotent Stem Cell Model
Source: Sci Rep. 2018 Feb 13;8:2942. doi: 10.1038/s41598-018-21103-8 (PMC5811493; doi:10.1038/s41598-018-21103-8)
Supplement: Supplementary file 1 — Supplementary Information [file 41598_2018_21103_MOESM1_ESM.pdf]

Folic Acid Exposure Rescues Spina Bifida Aperta Phenotypes in Human Induced Pluripotent Stem Cell Model

Vardine Sahakyan[1], Robin Duelen[1], Wai Long Tam[2], Scott J. Roberts[2, 3], Hanne Grosemans[1], Pieter Berckmans[4], Gabriele Ceccarelli[5], Gloria Pelizzo[6], Vania Broccoli[7, 8,] Jan Deprest[9], Frank P. Luyten[2], Catherine M. Verfaillie[4], Maurilio Sampaolesi[1, 5*]

[1]Translational Cardiomyology Laboratory, Stem Cell Biology and Embryology Unit, Stem Cell Institute, Department of Development and Regeneration, KU Leuven, Leuven, Belgium

[2]Tissue Engineering Laboratory, Skeletal Biology and Engineering Research Center, and Prometheus, Division of Skeletal Tissue Engineering, KU Leuven, Leuven, Belgium

[3]Institute of Orthopaedics and Musculoskeletal Science, Division of Surgery and Interventional Science, University College London, The Royal National Orthopaedic Hospital, London, United Kingdom

[4] Stem Cell Institute and Stem Cell Biology and Embryology Unit, Department Development and Regeneration, KU Leuven, Leuven, Belgium.

[5] Division of Human Anatomy, Department of Public Health, Experimental and Forensic Medicine, University of Pavia, Pavia, Italy.

[6] Department of Maternal and Children's Health, Pediatric Surgery Unit, Fondazione IRCCS Policlinico San Matteo and University of Pavia, Pavia Italy.

[7] San Raffaele Scientific Institute, Milan, Italy CNR-Institute of Neuroscience, Milan, Italy

[8] CNR-Institute of Neuroscience, Milan, Italy

[9] Department Development and Regeneration, Organ System Cluster and Group Biomedical Sciences and Division Woman and Child, University Hospitals Leuven, KU Leuven, Leuven, Belgium

[*] *Correspondence to* maurilio.sampaolesi@kuleuven.be

A

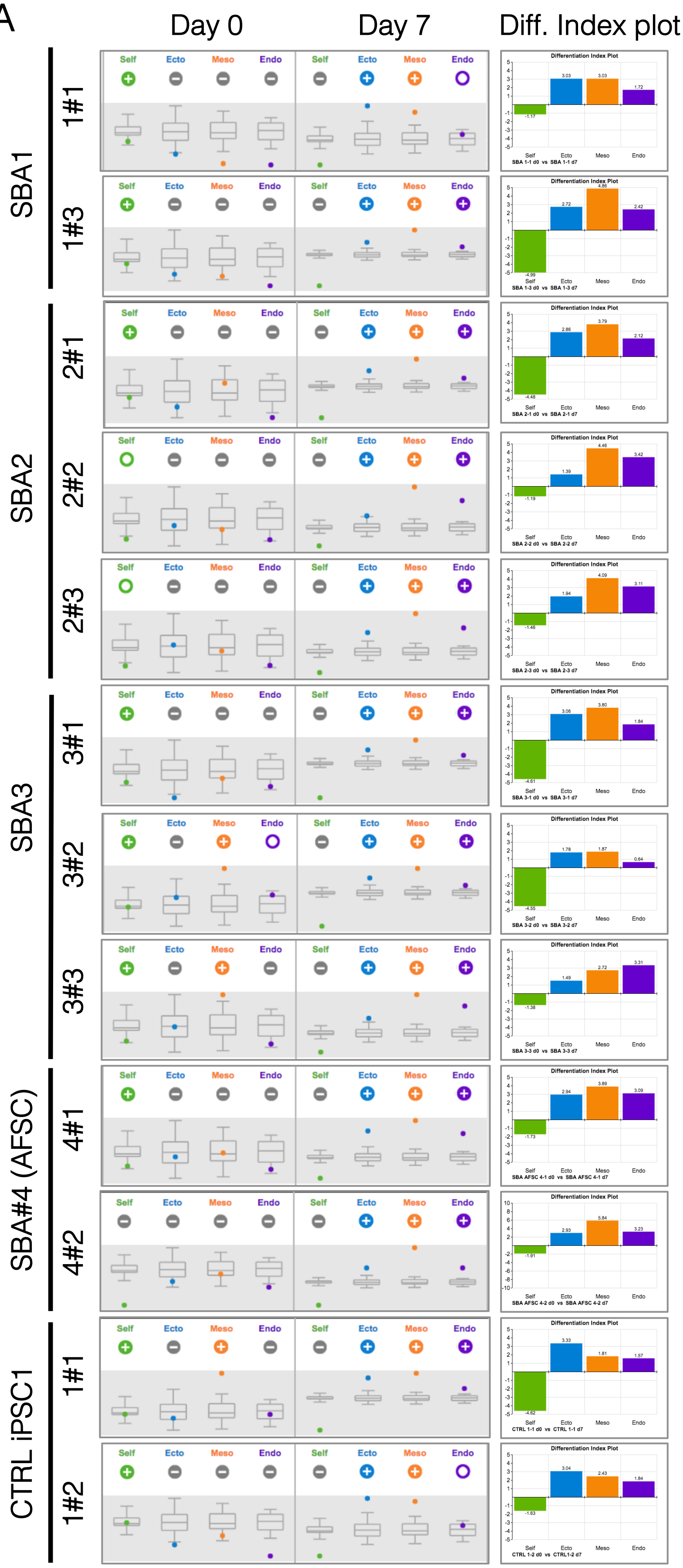

Day 0
Day 7
Diff. Index plot
SBA1
1#1
1#3
SBA2
2#1
2#2
2#3
SBA3
3#1
3#2
3#3
SBA#4 (AFSC)
4#1
4#2
CTRL iPSC1
1#1
1#2
Self
Ecto
Meso
Endo
Differentiation Index Plot

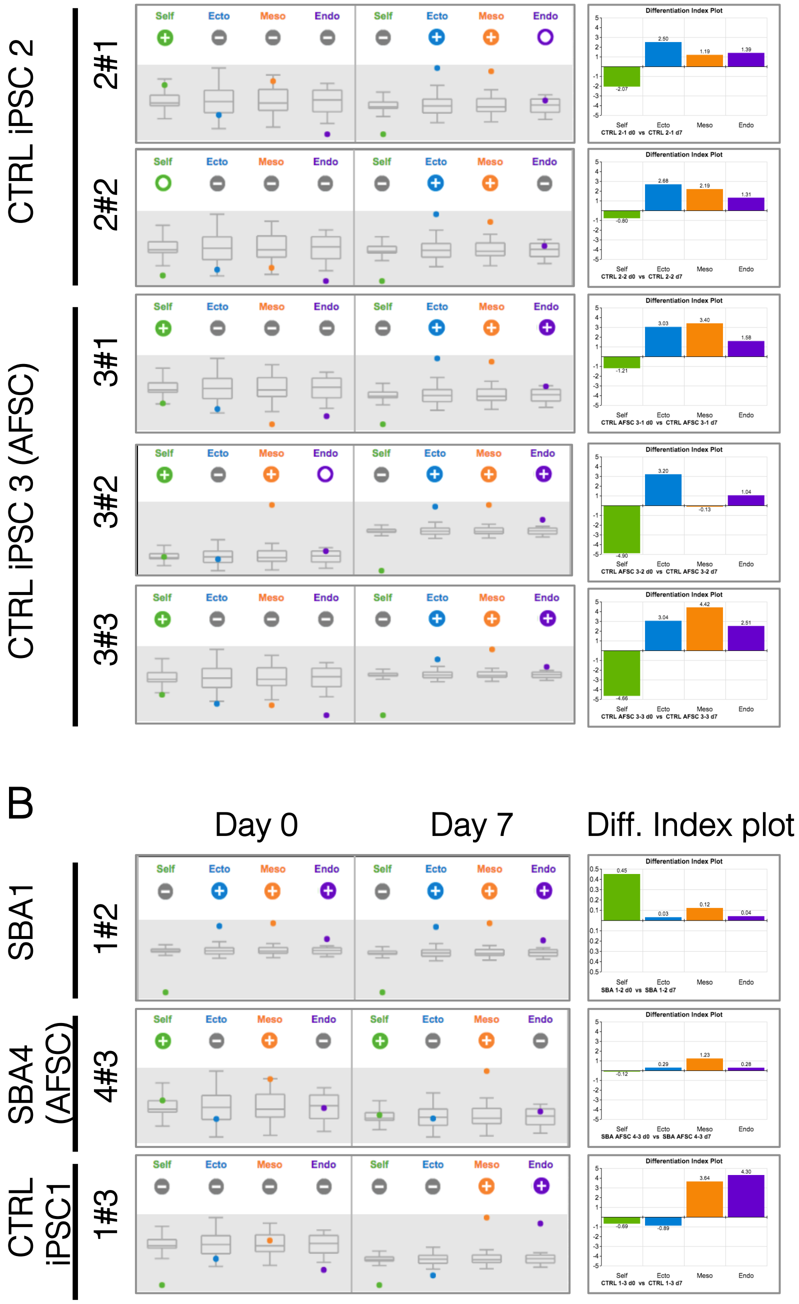

Supplementary Figure S1. ScoreCard analysis of pluripotency and *in vitro* differentiation potential of SBA and CTRL iPSC lines. (**A, B**) The left graph shows the results of hPSC ScoreCard for self-renewal (green), ectodermal (blue), mesodermal (orange) and endodermal (purple) differentiation potential of SBA and CTRL iPSC lines at day 0 (undifferentiated state) and at day 7 of spontaneous differentiation. The right graph shows the differentiation index plot at day 7 of spontaneous differentiation (downregulation of pluripotency and upregulation of three-germ layers representing markers) based on 94 predefined TaqMan Gene Expression assays (including endogenous controls).

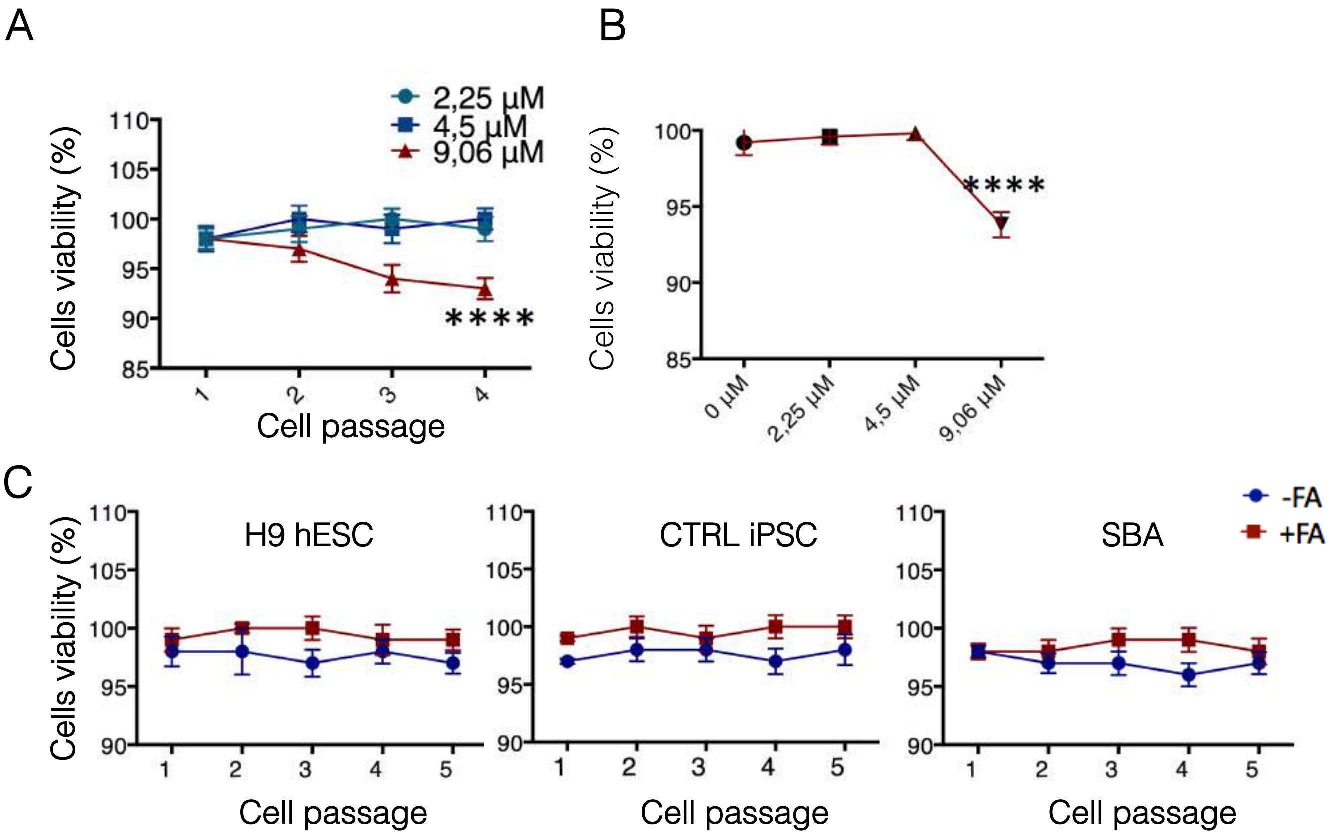

Supplementary Figure S2. Effect of FA exposure on cell viability. (**A**) FA dose dependent/viability assay on SBA lines, in 3 different concentrations shown as percentage. Data are plotted as average ± SEM. ****P <0.0001 2,25 µM *vs* 9,06 µM, 4,5 µM *vs* >9,06 µM FA. N=4 independent experiment per iPSC line. (**B**) FA Viability assay for SBA-derived NSCs, exposed to different concentrations of FA during the first 12 days of neural induction shown as percentage. Data are plotted as average ± SEM. ****P <0.0001 2,25 µM *vs* 9,06 µM, 4,5 µM *vs* >9,06 µM FA. N=4 independent experiment per iPSC line. (**C**) Viability assay on H9 hESCs, CTRL iPSC and SBA lines administrated or not with 4,5 µM FA during 5 passages shown as percentage. Data are plotted as average ± SEM. N=4 independent experiment per iPSC line.

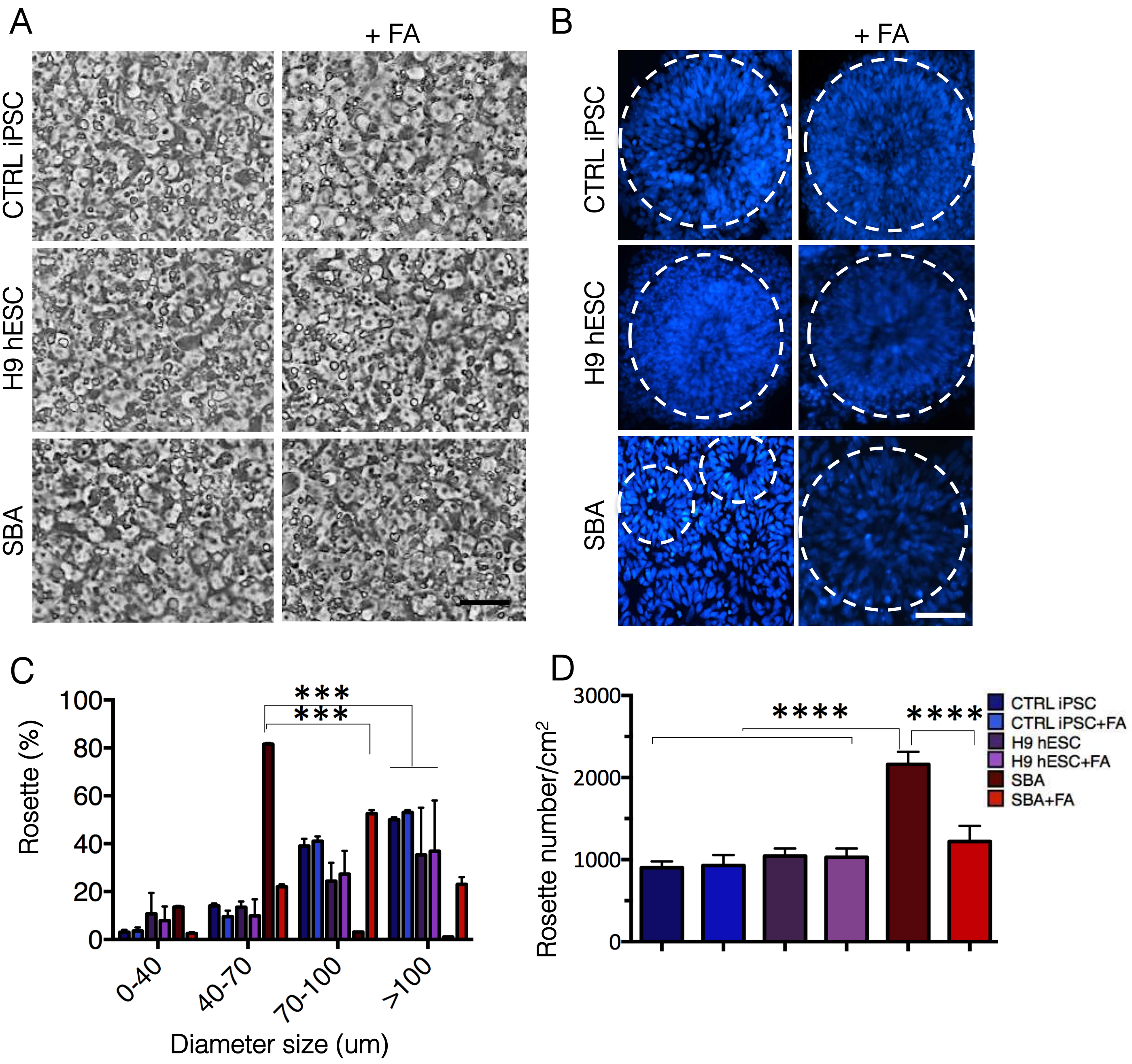

Supplementary Figure S3. Morphology of neural rosettes derived from SBA fetal iPSCs. (**A**) Microscope images of CTRL iPSC, H9 hESC and SBA-derived NSCs at day 6 of neural differentiation. Representative images of CTRL1#2 and SBA2#1 are shown. Scale bar = 100µm. (**B**) Examples of CTRL iPSC, H9 hESC and SBA-derived rosettes at day 18 of neural differentiation stained with DAPI. Representative images of CTRL1#2 and SBA2#1 are shown. Scale bar = 100µm. (**C**) Diameter size (µm) measurement of CTRL iPSC, H9 hESC and SBA-derived neural rosettes at day 18 of neural differentiation. Data are plotted as average ± SEM. ****P < 0.0001 SBA *vs* SBA+FA, ***P < 0.001 SBA *vs* CTRL iPSC and CTRL iPSC+FA, ***P <0,001 SBA *vs* H9 hESC and H9 hESC +FA. N=5 independent experiment per line. (**D**) Quantification of CTRL iPSC, H9 hESC and SBA-derived neural rosettes at day 18 of neural differentiation. Data are plotted as average ± SEM. ****P < 0.0001 H9 hESC, H9 hESC+FA, CTRL iPSC, CTRL iPSC+FA *vs* SBA, ****P < 0.0001 SBA *vs* SBA +FA. N=5 independent experiments per line.

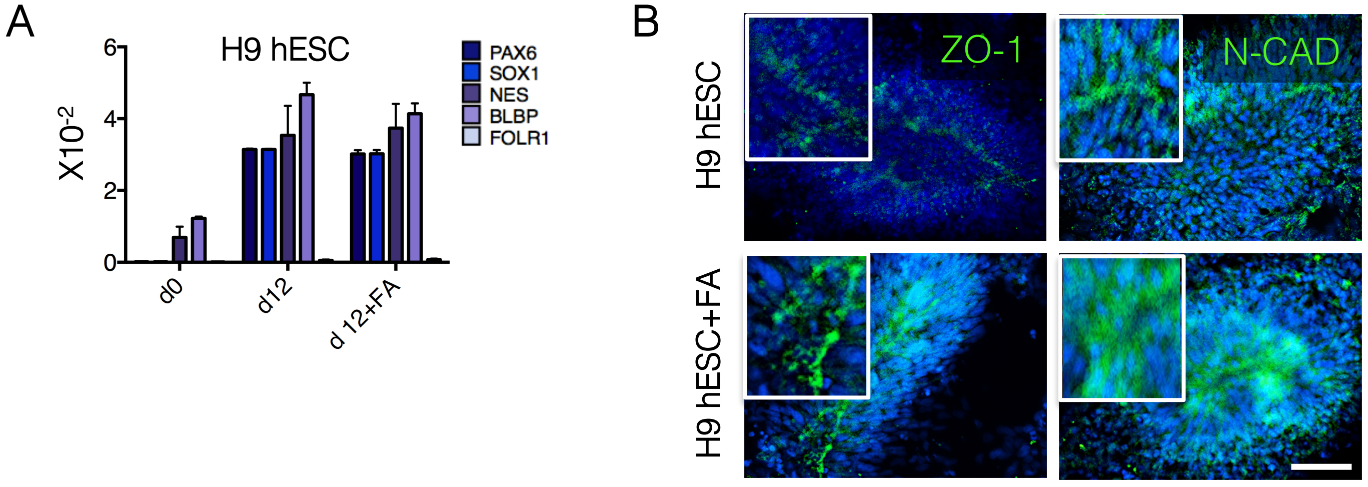

Supplementary Figure S4. Characterization of H9 hESC-derived neural rosettes. (A) qRT PCR data of H9 hESCs for pluripotency (*OCT4*), early neural (*PAX6*, *SOX1*, *NES),* radial glial (*BLBP)* and folate receptor 1 (*FOLR1*) genes at day 0 and day 12 of neural differentiation were shown as relative expression to house keeping gene *GAPDH*. NS, not significant. Data are plotted as average ± SEM. N=4 independent experiments. (**B**) IF staining for adherence and tight junction protein markers ZO-1 and N-CAD (both green) in H9 hESC-derived neural rosettes at day 18 of neural differentiation. Nuclei were counterstained with DAPI (blue). Scale bar = 100µm.

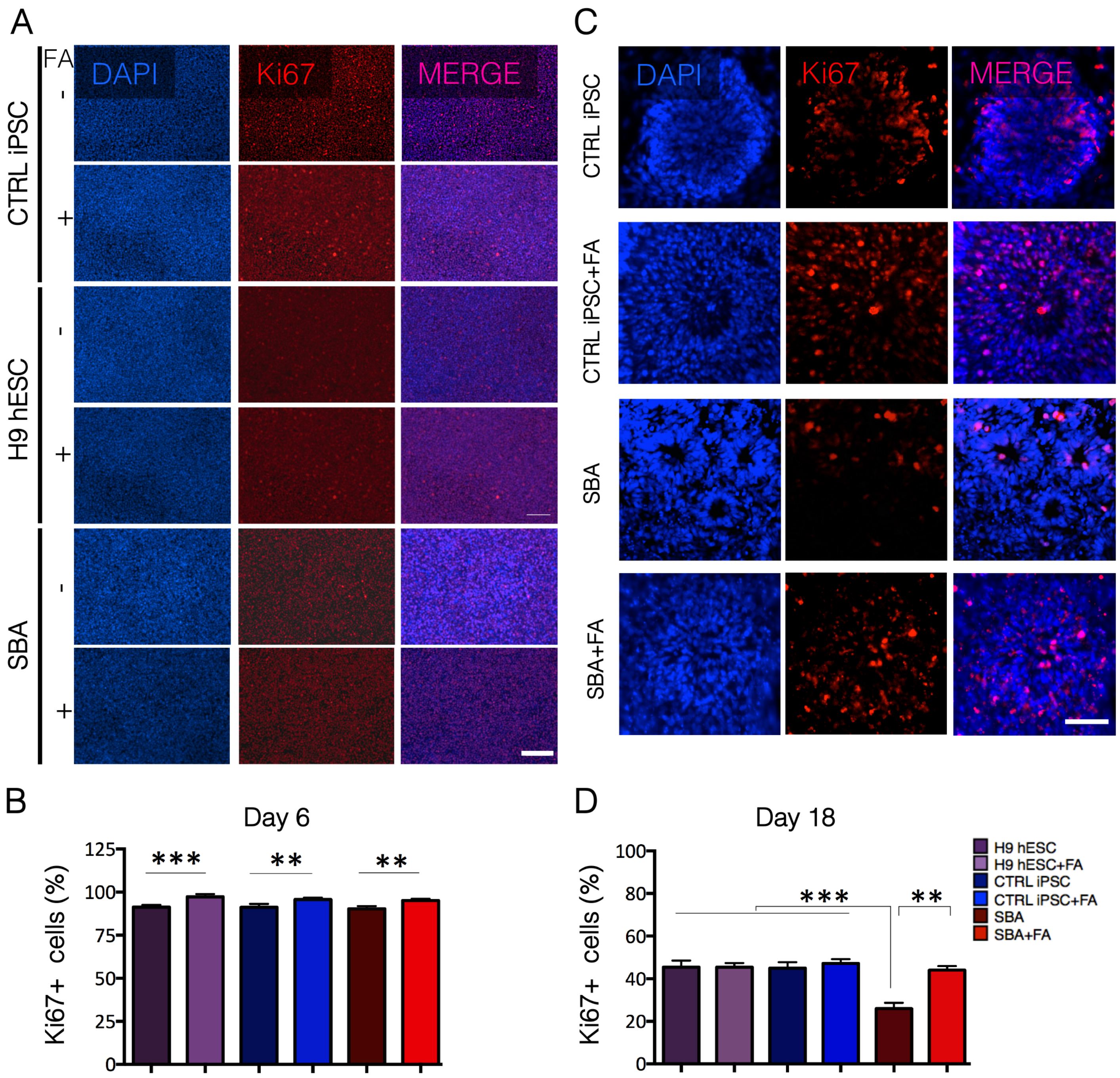

Supplementary Figure S5. Proliferation capacity of SBA iPSC-derived neural rosettes. (**A**) IF staining for Ki67 (red) in SBA, CTRL iPSC and H9 hESC-derived NSCs at day 6 of neural differentiation. Representative images of CTRL1#2 and SBA2#1 are shown. Nuclei were counterstained with DAPI (blue). Scale bar = 100µm. (**B**) Enumeration of Ki67-positive cells in CTRL iPSC, H9 hESC and SBA-derived NSCs at day 6 of neural differentiation shown as percentage. Data are plotted as average ± SEM. ***P <0.001 H9 hESC *vs* H9 hESC+FA, **P <0.01 CTRL iPSC *vs* CTRL iPSC+FA and SBA *vs* SBA+FA. N=4 independent experiment per line. (**C**) IF staining for Ki67 (red) in CTRL iPSC, H9 hESC and SBA-derived neural rosettes at day 18 of neural differentiation. Representative images of CTRL iPSC 3#3 (AFSC) and SBA2#1 are shown. Nuclei were counterstained with DAPI (blue). Scale bar = 100µm. (**D**) Enumeration of Ki67-positive cells in H9 hESC, CTRL iPSC and SBA-derived neural rosettes at day 18 of neural differentiation shown as percentage. Data are plotted as average ± SEM. ***P <0.001 H9 hESC, H9 hESC+FA, CTRL iPSC, CTRL iPSC+FA *vs* SBA and **P <0.01 SBA *vs* SBA+FA. N=4 independent experiment per line.

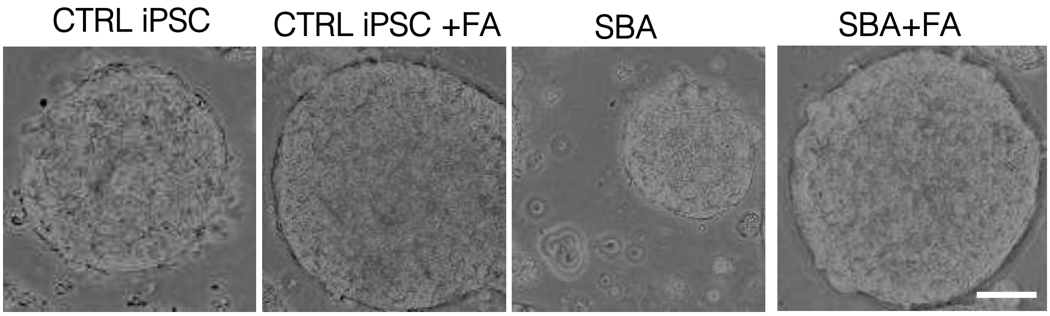

Supplementary Figure S6. Microscope images of spheroids generated from CTRL iPSC and SBA-derived neural rosettes. Representative images of CTRL1#2 and SBA2#1 are shown. Scale bar =200 µm.

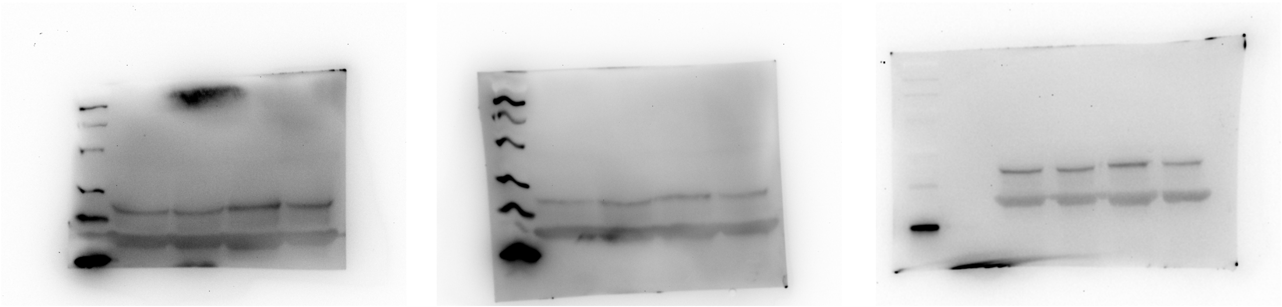

Supplementary Figure S7. Uncropped gels/blots for main Figure 7D,E.

Supplementary Table S1

Primers List

Gene	Forward primer	Reverse primer
GAPDH	TCAAGAAGGTGGTGAAGCAGG	ACCAGGAAATGAGCTTGACAAA
OCT4	CGAGCAATTTGCCAAGCTCCTGAA	GCCGCAGCTTACACATGTTCTTGA
SOX2	TGGCGAACCATCTCTGTGGT	CCAACGGTGTCAACCTGCAT
NANOG	TGGCCGAAGAATAGCAATGGTGTG	TTCCAGGTCTGGTTGCTCCACATT
cMYC	TCCTCGGATTCTCTGCTCTCCT	AGAAGGTGATCCAGACTCTGACCT
KLF4	CGGACATCAACGACGTGAG	GACGCCTTCAGCACGAACT
PAX6	GTGTCCAACGGATGTGTGAG	CTAGCCAGGTTGCGAAGAAC
SOX1	TACAGCCCCATCTCCAACTC	GCTCCGACTTCACCAGAGAG
BLBP	GGACTCTCAGCACATTCAAGAA	CCACATCACCAAAAGTAAGGGT
NES	CAGCGTTGGAACAGAGGTTGG	TGGCACAGGTGTCTCAAGGGTAG
BRN3A	GGGCAAGAGCCATCCTTTCAA	CTGTTCATCGTGTGGTACGTG
TBR1	ATGGGCAGATGGTGGTTTTA	GACGGCGATGAACTGAGTCT
RELN	GTAGCAAGCCCTTCAGCAAC	CCCTGAGGCCAGTACAACAT
DES	GAAGCTGCTGGAGGGAGAG	ATGGACCTCAGAACCCCTTT
PDGFRA	GATTAAGCCGGTCCCAACCT	GGATCTGGCCGTGGGTTT
PDGFRB	TGGCAGAAGAAGCCACGTT	GGCCGTCAGAGCTCACAGA
PAX3	GCCGCATCCTGAGAAGTAAA	CTTCATCTGATTGGGGTGCT
PAX7	GAGGATGAAGCGGACAAGAA	TCAGTGGGAGGTCAGGTT
MYH3	GCCCTTTGACATTCGCACTG	CGGGACAAAATCTTGGCTTTGA
COL2A1	GGCAATAGCAGGTTCACGTACA	CGATAACAGTCTTGCCCCACTT
RUNX2	CATGGTGGAGATCATCGC	ACTCTTGCCTCGTCCACTC
OCN	ATGAGAGCCCTCACACTCCTC	GCCGTAGAAGCGCCGATAGGC
SOX9	GACTTCCGCGACGTGGAC	GTTGGGCGGCAGGTACTG
ACAN	TCGAGGACAGCGAGGCC	TCGAGGGTGTAGCGTGTAGAGA
E-CAD	CGAACTATATTCTTCTGTGAGAGG	GATAGATTCTTGGGTTGGGTC
MIXL1	GGATCCAGGTATGGTTCCAG	CATGAGTCCAGCTTTGAACC
GATA4	TCCAAACCAGAAAACGGAAG	CTGTGCCCGTAGTGAGATGA
FOXA1	AGGCCTACTCCTCCGTCCCG	CTAGGCCCGGGTTGGCATAGG
DLX5	CGCCTCGCTGGGATTG	CTTGATCTTGGATCTTTTGTTCTGAA
SOX17	CGCTTTCATGGTGTGGGCTAAGGACG	TAGTTGGGGTGGTCCTGCATGTGCTG
HNF6	AAATCACCATTTCCCAGCAG	ACTCCTCCTTCTTGCGTTCA
FOLR1	GGACTGAGCTTCTCAATGTCT	GTCCTGGATGAAATGCCGTTT

Supplementary Table S2

Primary Antibodies

**Primary Antibody**	**Dilution**	**Cat Number**
OCT4	1:200	Santa Cruz, Sc-8628
SOX2	1:500	Santa Cruz, sc-17320
LIN28	1:500	Santa Cruz, sc-54032
TRA1-60	1:500	Santa Cruz, sc-21705
SSEA-4	1:500	Santa Cruz, sc-59368
NANOG	1:2000	Abcam, ab80892
PAX6	1:200	Covance, PRB-278P
NESTIN	1:200	Covance, MMS-570P
BLBP	1:2000	Chemicon, AB9558
NCAD	1:300	Abcam, ab18203
ZO1	1:300	Thermo Fisher scientific, PA5-21965
β III Tubulin	1:1000	Santa Cruz, sc-21705
αSMA	1:200	Sigma, C6198
AFP	1:800	Dako, A0008
PAX3	1:1000	R&D system, MAB2457
Ki67	1:50	Dako, M7240

Supplementary Note

iPSC lines used in the study

We employed in this study 7 and 10 individual cell colonies from CTRL and SBA iPSCs respectively. Each iPSC colony was tested for neural differentiation at least four times.

CTRL iPSC colonies:

2 individual colonies from each healthy (CTRL1, CTRL2) donor iPSC lines: CTRL1#1, CTRL1#2, CTRL2#1 and CTRL2#2; 3 clones from a healthy (CTRL3) donor iPSC line generated from amniotic fluid derived stem cells (CTRL3, AFSC): CTRL3#1, CTRL3#2 and CTRL3#3.

SBA iPSC colonies:

2-3 individual colonies from each iPSC line generated from 3 SBA fetal skin fibroblats (SBA1, 2 and 3):

SBA1#1 and SBA1#3; SBA2#1, SBA2#2 and SBA2#3 and SBA3#1, SBA3#2 and SBA3#3.

2 individual colonies from an iPSC line (SBA4) generated from SBA AFSCs: SBA4#1and SBA4#2.
